# A Bioconductor workflow for processing and analysing spatial proteomics data

**DOI:** 10.12688/f1000research.10411.2

**Published:** 2018-07-03

**Authors:** Lisa M. Breckels, Claire M. Mulvey, Kathryn S. Lilley, Laurent Gatto

**Affiliations:** 1 Computational Proteomics Unit, Cambridge Systems Biology Centre, University of Cambridge, Cambridge, UK; 2 Cambridge Centre for Proteomics, Department of Biochemistry, University of Cambridge, Cambridge, UK

**Keywords:** Bioconductor, R Package, proteomics, spatial proteomics, protein sub-cellular localisation, mass spectromery, machine learning, transfer learning

## Abstract

Spatial proteomics is the systematic study of protein sub-cellular localisation. In this workflow, we describe the analysis of a typical quantitative mass spectrometry-based spatial proteomics experiment using the *MSnbase* and *pRoloc* Bioconductor package suite. To walk the user through the computational pipeline, we use a recently published experiment predicting protein sub-cellular localisation in pluripotent embryonic mouse stem cells. We describe the software infrastructure at hand, importing and processing data, quality control, sub-cellular marker definition, visualisation and interactive exploration. We then demonstrate the application and interpretation of statistical learning methods, including novelty detection using semi-supervised learning, classification, clustering and transfer learning and conclude the pipeline with data export. The workflow is aimed at beginners who are familiar with proteomics in general and spatial proteomics in particular.

## Introduction

Quantitative mass spectrometry-based spatial proteomics involves elaborate, expensive and time consuming experimental protocols and considerable effort is invested in the generation of such data. Multiple research groups have described a variety of approaches to establish high quality proteome-wide datasets (see for example 1 for a review, and 2– 6 for recent examples). However, data analysis is as critical as data production for reliable and insightful biological interpretation. Here, we walk the reader through a typical pipeline for the analysis of such data using several Bioconductor ^7^ packages for the R statistical programming environment.

The main package to analyse protein localisation data is *pRoloc*, which offers a set of dedicated functions for the analysis of such data. *pRoloc* itself relies on *MSnbase* to manipulate and process quantitative proteomics data. Many other packages are used by *pRoloc* for clustering, classification and visualisation. Support for interactive visualisation is offered by the *pRolocGUI* package.

**Figure 1.**
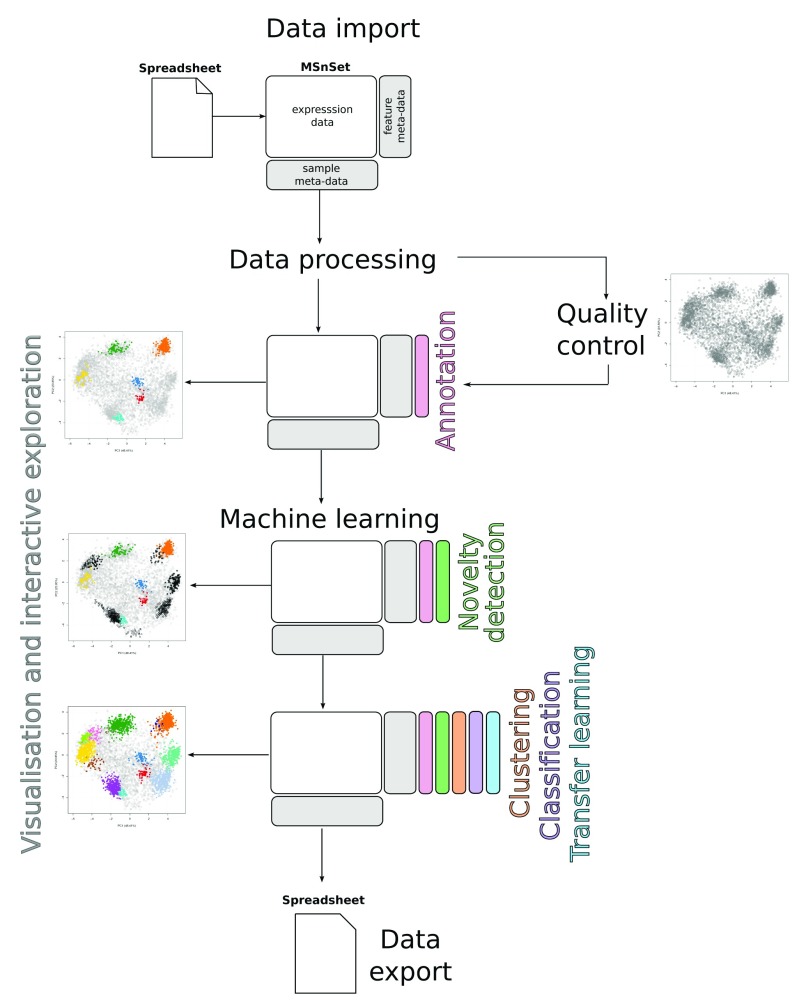
Schematic overview of the pRoloc pipeline from data import, through to data processing, machine learning and data export.

In this workflow, we will describe how to prepare the spatial proteomics data starting from a spreadsheet containing quantitative mass spectrometry data, through to some essential data processing steps, and finish with different applications of machine learning ( Figure 1). We focus on a recent pluripotent mouse embryonic stem cells experiment ^2^. These data, as well as additional annotated and pre-formatted datasets from various species are readily available in the *pRolocdata* package.

Installation of Bioconductor packages is documented in detail on the Bioconductor installation help page. Below, we show how to install the four main packages used in this workflow:


 source ( "https://bioconductor.org/biocLite.R" )
biocLite ( c ( "MSnbase" , "pRoloc" , "pRolocdata" , "pRolocGUI" ))
                


This procedure is also applicable to any packages, from CRAN as well as GitHub. Once a package has been installed, it needs to be loaded for its functionality to become available in the R session; this is done with the library function e.g. to load the *pRoloc* package one would type library(" pRoloc") after installation.

If you have questions about this workflow in particular, or about other Bioconductor packages in general, they are best asked on the Bioconductor support site following the posting guidelines. Questions can be tagged with specific package names or keywords. For more general information about mass spectrometry and proteomics, the readers are invited to read the *RforProteomics* package vignettes and associated papers ^8, 9^.

## Reading and processing spatial proteomics data

### The use-case: predicting sub-cellular localisation in pluripotent embryonic mouse stem cells

As a use-case, we analyse a recent high-throughput spatial proteomics dataset from pluripotent mouse embryonic stem cells (E14TG2a) ^2^. The data was generated using hyperplexed LOPIT (hyperLOPIT), a state-of-the-art method relying on improved sub-cellular fractionation and more accurate quantitation, leading to more reliable classification of protein localisation across the whole sub-cellular space. The method uses an elaborate sub-cellular fractionation scheme, enabled by the use of Tandem Mass Tag (TMT) ^10^ 10-plex and application of the MS data acquisition technique named synchronous precursor selection MS ^3^ (SPS-MS ^3^) ^11^, for TMT quantification with high accuracy and precision. Three biological replicates were generated from the E14TG2a experiment, the first was to target low density fractions and the second and third were to emphasis separation of the denser organelles. The intersect of replicates 1 and 2 was treated as a 20-plex dataset for the analysis. As discussed in the publication ^2^, it has been shown that combining replicates from different gradients can increase spatial resolution ^12^. The combination of replicates resulted in 5032 proteins common to both experiments.

These, as well as many other data are directly available as properly structured and annotated datasets from the *pRolocdata* experiment package. In this workflow, we will start with a description of how to generate these ad hoc data objects starting from an arbitrary spreadsheet, as produced by many popular third-party applications.

While we focus here on a LOPIT-type dataset, these analyses are relevent for any quantitative spatial proteomics data, irrespective of the fractionation (i.e. density gradient or differential centrifugation ^3^) or quantitation (i.e. labelled or label-free) methods.

### The infrastructure: *pRoloc* and *MSnbase* packages

**Figure 2.**
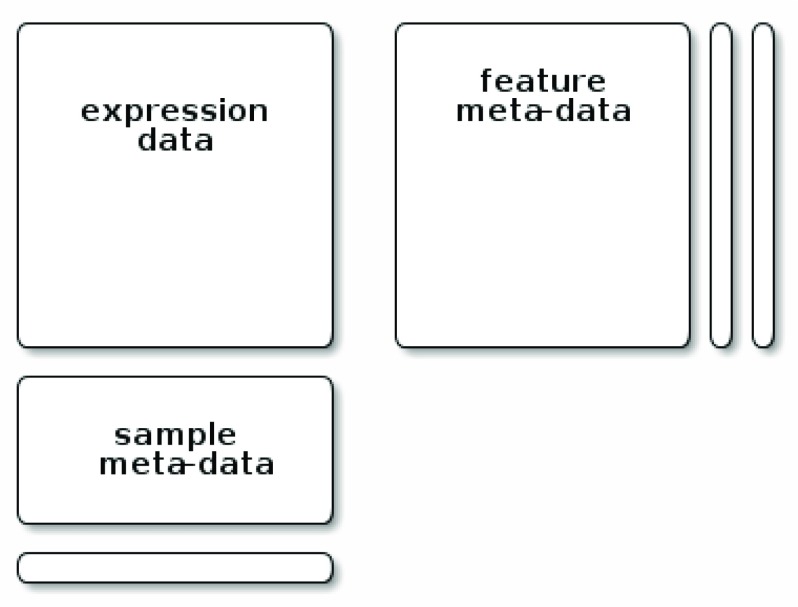
Simplified representation of the MSnSet data structure (reproduced with permission from the *MSnbase* vignette).

To make use of the full functionality of the *pRoloc* software, users need to import their data into R and prepare them as an MSnSet. The MSnSet is a dedicated data structure for the efficient manipulation and processing of mass spectrometry and proteomics data in R. Figure 2 illustrates a simplified view of the MSnSet structure; there exists 3 key sub-parts (termed slots) to such a data object: (1) the exprs (short for *expression* data) slot for storing the quantitation data, (2) the fData slot (short for *feature*-metadata) for storing the feature meta-data, and finally (3) the pData slot (short for *pheno*-metadata, i.e. sample phenotypic data) for storing the sample meta-data.

Feature metadata typically contains general annotation about the proteins (accession numbers, description, …), information related to the identification search (confidence scores, number of peptides, …) as well as annotation about known sub-cellular location (see in particular the *Markers* section) and results from data analysis. The sample metadata would, for example, record what stable isotope labels were used for the respective fraction (when labelled quantitation is used), replicate number, fraction number along the gradient and pooling information.

Another slot of interest is processingData, that logs the processing MSnSet objects undergo. The processing log can be accessed with the processingData function and is displayed under *Processing information* in the textual object summary when an MSnSet’s name is typed in the R console (see example below).

### Importing data

There are a number of ways to import quantitation data and create an MSnSet instance. All methods are described in the *MSnbase* input/output capabilities vignette. The simplest method is to use the function readMSnSet2. The function takes a single spreadsheet file name as input and extracts the columns containing the quantitation data, as identified by the argument ecol, to create the expression data, while the other columns in the spreadsheet are appended to the feature meta-data slot. By example, in the code chunk below we read in the csv spreadsheet containing the quantitation data from the intersect of replicates 1 and 2 of the mouse map ^2^, using the readMSnSet2 function. The data is as available online with the manuscript (see tab 2 of the xlsx supplementary data set 1 in 2, which should be exported as a text-based spreadsheet). It is also available as a csv in the Bioconductor *pRolocdata* data package, which we make use of below.

To use the readMSnSet2 function, as a minimum one must specify the file path to the data and which columns of the spreadsheet contain quantitation data. In the code chunk below, we start by identifying the file that we want to use. The system.file function is used to return the path to the extdata directory from the *pRolocdata* package, which is where our file of interest resides. We then use the dir function to list the content of that directory and store the path that matches the file name of interest in the csvfile variable. Note that these two lines are only needed here to locate a file in a package; in a more general use case, the user would define the csvfile variable containing the file name of interest directly.

A common pitfall here is to provide only the file name, rather than full path to the file (which is what is shown below with basename; we don’t print the full path, as it will vary from computer to computer). Note that only specifying the name of the file is sufficient when it exists in the working directory (i.e. the directory in which R is running, which can be queried and changed with the getwd and setwd functions respectively).




extdatadir <- system.file ( "extdata" , package = "pRolocdata" )

csvfile <- dir (extdatadir, full.names = TRUE ,
         pattern = "hyperLOPIT-SIData-ms3-rep12-intersect.csv" )

basename (csvfile)


## [1] "hyperLOPIT-SIData-ms3-rep12-intersect.csv.gz"
                    


Note that the file is compressed (as indicated by the gz, for gzip, extension), and will be decompressed on-the-fly when read into R.

**Figure 3.**
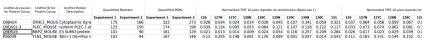
A screenshot of the data in the spreadsheet.

Next, we need to identify which columns in the spreadsheet contain the quantitation data. This can be done using the getEcols function inside R. The spreadsheet deposited by the authors contains two headers, with the second header containing information about where the quantitation data is stored ( Figure 3).

We can display the names of the second header by calling the getEcols function with the argument n = 2 (the default value is n = 1), to specify that we wish to display the column names of the second line. We also specify the name of the spreadsheet file (defined as csvfile above) and the separator that splits cells.



getEcols (csvfile, split = "," , n = 2 )


##  [1] ""
##  [2] ""
##  [3] ""
##  [4] "Experiment 1"
##  [5] "Experiment 2"
##  [6] "Experiment 1"
##  [7] "Experiment 2"
##  [8] "126"
##  [9] "127N"
## [10] "127C"
## [11] "128N"
## [12] "128C"
## [13] "129N"
## [14] "129C"
## [15] "130N"
## [16] "130C"
## [17] "131"
## [18] "126"
## [19] "127N"
## [20] "127C"
## [21] "128N"
## [22] "128C"
## [23] "129N"
## [24] "129C"
## [25] "130N"
## [26] "130C"
## [27] "131"
## [28] "phenoDisco Input"
## [29] "phenoDisco Output"
## [30] "Curated phenoDisco Output"
## [31] "SVM marker set"
## [32] "SVM classification"
## [33] "SVM score"
## [34] "SVM classification (top quartile)"
## [35] "Final Localization Assignment"
## [36] "First localization evidence?"
## [37] "Curated Organelles"
## [38] "Cytoskeletal Components"
## [39] "Trafficking Proteins"
## [40] "Protein Complexes"
## [41] "Signaling Cascades"
## [42] "Oct4 Interactome"
## [43] "Nanog Interactome"
## [44] "Sox2 Interactome"
## [45] "Cell Surface Proteins"
                    


It is now easy for one to identify that the quantitation data, corresponding to the 10 TMT isobaric tags, is located in columns 8 to 27. We now have the two mandatory arguments to readMSnSet2, namely the file name (stored in the csvfile variable) and the quantitation column indices. In addition to these, it is also possible to pass the optional argument fnames to indicate which column to use as the labels by which to identify each protein in the sample. Here, we use fnames = 1 to use the UniProt identifiers contained in the first (unnamed) column of the spreadsheet. We also need to specify to skip the first line of the file (for the same reason that we used n = 2 in getEcols above) to read the csv data and convert it to an MSnSet object, named hl (for hyperLOPIT).



hl <- readMSnSet2 (csvfile, ecol = 8: 27 , fnames = 1 , skip = 1 )
                    


Below, we display a short summary of the data. The data contains 5032 proteins/features common across the 2 biological replicates for the respective 2 × 10-plex reporter tags (20 columns or samples), along with associated feature meta-data such as protein markers, protein description, number of quantified peptides etc (see below).



hl


## MSnSet (storageMode: lockedEnvironment)
## assayData: 5032 features, 20 samples
##   element names: exprs
## protocolData: none
## phenoData: none
## featureData
##   featureNames: Q9JHU4 Q9QXS1-3 ... Q9Z2R6 (5032 total)
##   fvarLabels: X X.1 ... Cell.Surface.Proteins (25 total)
##   fvarMetadata: labelDescription
## experimentData: use 'experimentData(object)'
## Annotation:
## - - - Processing information - - -
##  MSnbase version: 2.7.1
                    


Below, we examine the quantitative information along the whole gradient for first 5 proteins. It is also possible to access specific rows and columns by naming the proteins and TMT tag channels of interest.



exprs (hl)[ 1: 5 , ]

##           X126 X127N X127C X128N X128C X129N X129C X130N X130C  X131
## Q9JHU4   0.028 0.034 0.024 0.014 0.026 0.045 0.107 0.341 0.059 0.321
## Q9QXS1-3 0.039 0.134 0.095 0.053 0.084 0.121 0.107 0.128 0.122 0.117
## Q9ERU9   0.021 0.013 0.014 0.009 0.024 0.054 0.116 0.257 0.209 0.284
## P26039   0.120 0.255 0.148 0.091 0.135 0.095 0.041 0.057 0.014 0.043
## Q8BTM8   0.055 0.139 0.078 0.050 0.077 0.098 0.093 0.171 0.079 0.160
##          X126.1 X127N.1 X127C.1 X128N.1 X128C.1 X129N.1 X129C.1 X130N.1
## Q9JHU4    0.037   0.064   0.058   0.059   0.067   0.078   0.140   0.208
## Q9QXS1-3  0.033   0.073   0.074   0.062   0.081   0.142   0.190   0.069
## Q9ERU9    0.026   0.017   0.023   0.029   0.039   0.071   0.105   0.171
## P26039    0.111   0.181   0.141   0.144   0.152   0.119   0.075   0.028
## Q8BTM8    0.062   0.108   0.091   0.086   0.099   0.111   0.117   0.095
##          X130C.1 X131.1
## Q9JHU4     0.141  0.147
## Q9QXS1-3   0.151  0.125
## Q9ERU9     0.304  0.215
## P26039     0.017  0.033
## Q8BTM8     0.144  0.087


exprs (hl)[ c ( "Q9ERU9" , "Q9Z2R6" ), c ( "X126" , "X131.1" )]


##         X126 X131.1
## Q9ERU9 0.021  0.215
## Q9Z2R6 0.563  0.000
                    


The feature meta-data is stored in the fData slot and can be accessed by fData(hl). When using readMSnSet2 everything that is not defined as quantitation data by ecol is deposited to the fData slot.

We see the fData contains 25 columns describing information such as the number of peptides, associated markers, machine learning results etc. To identify the feature variable names we can use the function fvarLabels. We see that the first 6 feature variable names contain non-discriminatory label names, so we relabel them to help us identify what feature data information is stored in the associated columns.



fvarLabels (hl)


##  [1] "X"
##  [2] "X.1"
##  [3] "X.2"
##  [4] "Experiment.1"
##  [5] "Experiment.2"
##  [6] "Experiment.1.1"
##  [7] "Experiment.2.1"
##  [8] "phenoDisco.Input"
##  [9] "phenoDisco.Output"
## [10] "Curated.phenoDisco.Output"
## [11] "SVM.marker.set"
## [12] "SVM.classification"
## [13] "SVM.score"
## [14] "SVM.classification..top.quartile."
## [15] "Final.Localization.Assignment"
## [16] "First.localization.evidence."
## [17] "Curated.Organelles"
## [18] "Cytoskeletal.Components"
## [19] "Trafficking.Proteins"
## [20] "Protein.Complexes"
## [21] "Signaling.Cascades"
## [22] "Oct4.Interactome"
## [23] "Nanog.Interactome"
## [24] "Sox2.Interactome"
## [25] "Cell.Surface.Proteins"


fvarLabels (hl)[ 1: 3 ] <- c ( "uniprot.accession" , "uniprot.id" , "description" )
fvarLabels (hl)[ 4: 6 ] <- paste0 ( "peptides.expt" , 1: 3 )
## feature vars 1, 2, and 4 to 6
fData (hl)[ 1: 4 , c ( 1: 2 , 4: 6 )]


##          uniprot.accession  uniprot.id peptides.expt1 peptides.expt2
## Q9JHU4              Q9JHU4 DYHC1_MOUSE            175            166
## Q9QXS1-3          Q9QXS1-3  PLEC_MOUSE            123            150
## Q9ERU9              Q9ERU9  RBP2_MOUSE            101             90
## P26039              P26039  TLN1_MOUSE            101             94
##          peptides.expt3
## Q9JHU4              322
## Q9QXS1-3            174
## Q9ERU9              181
## P26039              167
                    


Note that when using the simple readMSnSet2 procedure, the pData slot which is used to store information about the samples/channels is kept empty. As illustrated below, one can use the $ operator to access (or create) individual columns in the metadata slot. It is advised to annotate the channels as well. Below, we annotate the replicate from which the profiles originate and the TMT tag (extracted from the sample/channel names). To do so, we use the sample names that were assigned automatically using the quantiation column names and remove leading X and trailing .1 using the sub function.



pData (hl)$ Replicate <- rep ( 1: 2 , each = 10 )
pData (hl)$ Tag <- sub ( "\\.1$" , "" , sub ( "^X" , "" , sampleNames (hl)))
pData (hl)


##         Replicate  Tag
## X126            1  126
## X127N           1 127N
## X127C           1 127C
## X128N           1 128N
## X128C           1 128C
## X129N           1 129N
## X129C           1 129C
## X130N           1 130N
## X130C           1 130C
## X131            1  131
## X126.1          2  126
## X127N.1         2 127N
## X127C.1         2 127C
## X128N.1         2 128N
## X128C.1         2 128C
## X129N.1         2 129N
## X129C.1         2 129C
## X130N.1         2 130N
## X130C.1         2 130C
## X131.1          2  131
                    


Throughout this workflow we refer to the different columns that are found in the exprs (expression data) slot as channels (short for TMT channels). In the frame of LOPIT and hyperLOPIT these channels constitute the relative abundance of each protein (along the rows) in the channel of interest. Each TMT channel originates from fractions collected from the density gradient, or a set of pooled fractions or may be a sample originating from an alternative preparation e.g. such as from the chromatin enrichment performed in Christoforou *et al.* ^2^ Information about which gradient fractions were used for which tag should also be stored in the sample meta-data pData slot.

The sample meta-data that is distributed with the *pRolocdata* package for Christoforou’s hyperLOPIT experiment and (as above) the quantitation data file, are located in the extdata in the *pRolocdata* package on the hard drive.

In the code chunk below we again use the dir function to locate the filepath to the meta-data csv file and then read it into R using read.csv. We then append the meta-data to the pData slot. Information about the gradient fractions used and the associated subcellular fraction densities in each replicate are stored here.



expinfo <- dir (extdatadir, full.names = TRUE ,
         pattern = "hyperLOPIT-SIData-fraction-info.csv" )

fracinfo <- read.csv (expinfo, row.names= 1 , skip = 2 ,
         header = FALSE , stringsAsFactors = FALSE )
pData (hl)$ Gradient.Fraction <- c (fracinfo[, 1 ], fracinfo[, 2 ])
pData (hl)$ Iodixonal.Density <- c (fracinfo[, 4 ], fracinfo[, 5 ])
pData (hl)


##         Replicate  Tag Gradient.Fraction Iodixonal.Density
## X126            1  126           Cytosol               0.0
## X127N           1 127N   1 to 6 (pooled)               6.0
## X127C           1 127C   8 to 9 (pooled)              11.0
## X128N           1 128N 10 to 11 (pooled)              13.3
## X128C           1 128C                12              14.6
## X129N           1 129N                14              17.4
## X129C           1 129C                16              20.1
## X130N           1 130N                18              26.8
## X130C           1 130C         Chromatin                NA
## X131            1  131                19              34.5
## X126.1          2  126           Cytosol               0.0
## X127N.1         2 127N   1 to 6 (pooled)               5.2
## X127C.1         2 127C   7 to 9 (pooled)              10.0
## X128N.1         2 128N 10 to 11 (pooled)              12.5
## X128C.1         2 128C                12              14.0
## X129N.1         2 129N 14 to 15 (pooled)              17.3
## X129C.1         2 129C                17              20.9
## X130N.1         2 130N 18 to 19 (pooled)              24.7
## X130C.1         2 130C         Chromatin                NA
## X131.1          2  131                20              31.9
                    


### Data processing

***Normalisation.*** There are two aspects related to data normalisation that are relevent to spatial proteomics data processing. The first one focuses on reducing purely technical variation between channels without affecting biological variability (i.e. the shape of the quantitatives profiles). This normalisation will depend on the underlying quantitative technology and the experimental design, and will not be addressed in this workflow. The second aspect, and more specific to spatial proteomics data, is scaling all the organelle-specific profiles into the same intensity interval (typically 0 and 1) by, for example, dividing each intensity by the sum of the intensities for that quantitative feature. This is not necessary in this example as the intensities for each replicate have already been re-scaled to 1 in Proteome Discoverer v1.4 Thermo Fisher. However, if the data require normalisation, the user can execute the normalise function as demonstrated in the below code chunk.



hl <- normalise (hl, method = "sum" )
                    


This transformation of the data assures cancellation of the effect of the absolute intensities of the quantitative features along the rows, and focus subsequent analyses on the relative profiles along the sub-cellular channels.

The same normalise function (or normalize, both spellings are supported) can also be applied in the first case described above. Different normalisation methods, such as mean or median scaling, variance stabilisation or quantile normalisation, to cite a few, can be applied to accomodate different needs (see ?normalise for available options).

As previously mentioned, before combination, the two replicates in the hl data that we read into R were separately normalised by sum (i.e. to 1) across the 10 channels for each replicate respectively. We can verify this by summing each rows for each replicate:



summary ( rowSums ( exprs (hl[, hl$ Replicate == 1 ])))


##    Min. 1st Qu.  Median  Mean 3rd Qu.  Max.
##   0.997   0.999   1.000 1.000   1.001 1.003


summary ( rowSums ( exprs (hl[, hl$ Replicate == 2 ])))


##    Min. 1st Qu.  Median  Mean 3rd Qu.  Max.
##   0.997   0.999   1.000 1.000   1.001 1.003
                    


We see that some features do not add up exactly to 1 due to rounding errors after exporting to intermediate files. These small deviations do not bear any consequences here.

### Combining acquisitions

The spreadsheet that was used to create the hl MSnSet included the two replicates within one .csv file. We also provide individual replicates in the *pRolocdata* package. Below, we show how to combine MSnSet objects and, subsequently, how to filter and handle missing values. We start by loading the *pRolocdata* package and the equivalent replicates using the data function.



library ( "pRolocdata" )
data (hyperLOPIT2015ms3r1)
data (hyperLOPIT2015ms3r2)
                    


At the R prompt, typing



pRolocdata ()
                    


will list the 75 datasets that are available in *pRolocdata*.

Combining data is performed with the combine function. This function will inspect the feature and sample names to identify how to combine the data. As we want our replicates to be combined along the columns (same proteins, different sets of channels), we need to assure that the respective sample names differ so they can be identified from one another. The function updateSampleNames can be used do this.



identical ( sampleNames (hyperLOPIT2015ms3r1), sampleNames (hyperLOPIT2015ms3r2))


## [1] TRUE


hyperLOPIT2015ms3r1 <- updateSampleNames (hyperLOPIT2015ms3r1, 1 )

hyperLOPIT2015ms3r2 <- updateSampleNames (hyperLOPIT2015ms3r2, 2 )

sampleNames (hyperLOPIT2015ms3r1)


## [1] "X126.1"  "X127N.1" "X127C.1" "X128N.1" "X128C.1" "X129N.1" "X129C.1"
## [8] "X130N.1" "X130C.1" "X131.1"


sampleNames (hyperLOPIT2015ms3r2)


## [1] "X126.2"  "X127N.2" "X127C.2" "X128N.2" "X128C.2" "X129N.2" "X129C.2"
## [8] "X130N.2" "X130C.2" "X131.2"
                    


In addition to matching names, the content of the feature metadata for identical feature annotations must match exactly across the data to be combined. In particular for these data, we expect the same proteins in each replicate to be annotated with the same UniProt entry names and descriptions, but not with the same coverage of number of peptides or peptide-spectrum matches (PSMs).



fvarLabels (hyperLOPIT2015ms3r1)


## [1] "EntryName"          "ProteinDescription" "Peptides"
## [4] "PSMs"               "ProteinCoverage"    "markers"


fvarLabels (hyperLOPIT2015ms3r2)


## [1] "EntryName"          "ProteinDescription" "Peptides"
## [4] "PSMs"               "ProteinCoverage"    "markers"
                    


Below, we update the replicate specific feature variable names and remove the shared annotation. In the first line, we update only the feature variable names 3 to 5 (by appending a 1) of the first replicate and in the second line, we apply the updateFvarLabels function to update all feature variable names (by appending a 2) of the second replicate. In lines 3 and 4, we retain the first 5 feature variables for the first replicate and the relevant third to fifth variables for the second replicate.



fvarLabels (hyperLOPIT2015ms3r1)[ 3: 5 ] <- paste0 ( fvarLabels (hyperLOPIT2015ms3r1)[ 3: 5 ], 1 )

hyperLOPIT2015ms3r2 <- updateFvarLabels (hyperLOPIT2015ms3r2, "2" , sep = "" )

fData (hyperLOPIT2015ms3r1) <- fData (hyperLOPIT2015ms3r1)[ 1: 5 ]

fData (hyperLOPIT2015ms3r2) <- fData (hyperLOPIT2015ms3r2)[ 3: 5 ]

fvarLabels (hyperLOPIT2015ms3r1)


## [1] "EntryName"          "ProteinDescription" "Peptides1"
## [4] "PSMs1"              "ProteinCoverage1"


fvarLabels (hyperLOPIT2015ms3r2)


## [1] "Peptides2"          "PSMs2"          "ProteinCoverage2"
                    


We can now combine the two experiments into a single MSnSet:



combined <- combine (hyperLOPIT2015ms3r1, hyperLOPIT2015ms3r2)

combined


## MSnSet (storageMode: lockedEnvironment)
## assayData: 6725 features, 20 samples
##   element names: exprs
## protocolData: none
## phenoData
##   sampleNames: X126.1 X127N.1 ... X131.2 (20 total)
##   varLabels: Replicate TMT.Reagent ... Iodixonal.Density (5 total)
##   varMetadata: labelDescription
## featureData
##   featureNames: Q9JHU4 Q9QXS1-3 ... Q9Z2Y3-3 (6725 total)
##   fvarLabels: EntryName ProteinDescription ... ProteinCoverage2 (8
##     total)
##   fvarMetadata: labelDescription
## experimentData: use 'experimentData(object)'
##   pubMedIds: 26754106
## Annotation:
## - - - Processing information - - -
## Combined [5489,10] and [6268,10] MSnSets Tue May 22 16:07:39 2018
##  MSnbase version: 2.5.9
                    


More details about combining data are given in the dedicated *Combining MSnSet instances* section of the *MSnbase* tutorial vignette.

### Missing data

Missing data are a recurrent issue in mass spectrometry applications, and should be addressed independently of this workflow ^13, 14^. In 15, we have described how a high content in missing values in spatial proteomics data and their inappropriate handling leads to a reduction of sub-cellular resolution. We can impute missing data using *MSnbase*’s impute function. The method underlying the imputation method is then determined by a methods parameter (see ?impute for available options). To impute missing values using nearest neighbour imputation, one would



hl <- impute (hl, method = "knn" )
                    


**Figure 4.**
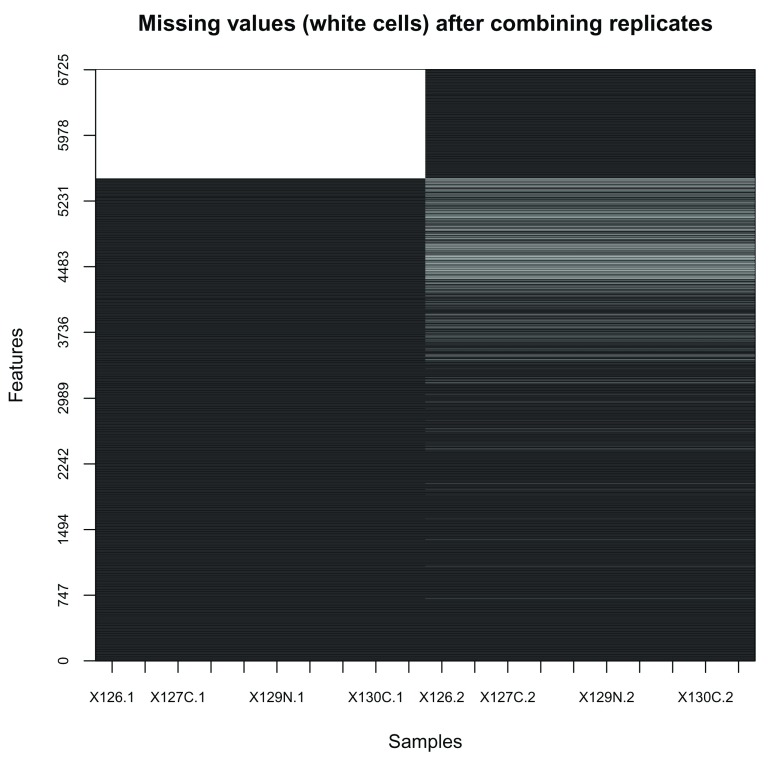
Heatmap of missing values. Note that the features are re-ordered to highlight clusters of proteins with similar numbers of missing values.

In our particular case, missing values are indicative of protein groups that were not acquired in both replicates ( Figure 4, produced with the image2 function).



image2 ( is.na (combined), col = c ( "black" , "white" ),
        main = "Missing values (white cells) after combining replicates" )
                    


We prefer to remove proteins that were not assayed in both replicated experiments. This is done with the filterNA function that removes features (proteins) that contain more than a certain proportion (default is 0) missing values. The *Processing information* section summarises how combining and filtering missing values (subsetting) changed the dimensions of the data.



combined <- filterNA (combined)

combined


## MSnSet (storageMode: lockedEnvironment)
## assayData: 5032 features, 20 samples
##   element names: exprs
## protocolData: none
## phenoData
##   sampleNames: X126.1 X127N.1 ... X131.2 (20 total)
##   varLabels: Replicate TMT.Reagent ... Iodixonal.Density (5 total)
##   varMetadata: labelDescription
## featureData
##   featureNames: Q9JHU4 Q9QXS1-3 ... Q9Z2R6 (5032 total)
##   fvarLabels: EntryName ProteinDescription ... ProteinCoverage2 (8
##     total)
##   fvarMetadata: labelDescription
## experimentData: use 'experimentData(object)'

                    




##   pubMedIds: 26754106
## Annotation:
## - - - Processing information - - -
## Combined [5489,10] and [6268,10] MSnSets Tue May 22 16:07:39 2018
## Subset [6725,20][5032,20] Tue May 22 16:07:40 2018
## Removed features with more than 0 NAs: Tue May 22 16:07:40 2018
## Dropped featureData's levels Tue May 22 16:07:40 2018
##  MSnbase version: 2.5.9
                    


When more than 2 datasets are to be combined and too many proteins have not been consistently assayed, leading to too many proteins being filtered out, we suggest to implement an ensemble of classifiers voting on protein-sub-cellular niche membership over the output of several experiments (see section *Supervised machine learning* for the description of sub-cellular assignments).

## Quality control

Data quality is routinely examined through visualisation to verify that sub-cellular niches have been separated along the gradient. Based on De Duve's principle ^16^ proteins that co-localise in a cell, exhibit similar quantitation profiles across the gradient fractions employed. One approach that has been widely used to visualise and inspect high throughput mass spectrometry-based proteomics data is principal components analysis (PCA). PCA is one of many dimensionality reduction methods, that allows one to effectively summarise multi-dimensional data in to 2 or 3 dimensions to enable visualisation. Very generally, the original continuous multi-dimensional data is transformed into a set of orthogonal components ordered according to the amount of variability that they describe. The plot2D and plot3D functions in *pRoloc* allows one to plot the principal components (PCs) of a dataset against one another. By default, the first two components are plotted on the x- and y-axis for the plot2D function, and first three components are loaded for the plot3D function, respectively (the dims argument can be used to plot other PCs). If distinct clusters are observed, we assume that there is organellar separation present in the data. Although, representing the multi-dimensional data along a limited set of PCs does not give us a hard quantitative measure of separation, it is extremely useful summarising complex experimental information in one figure, to get a simplified overview of the data.

**Figure 5.**
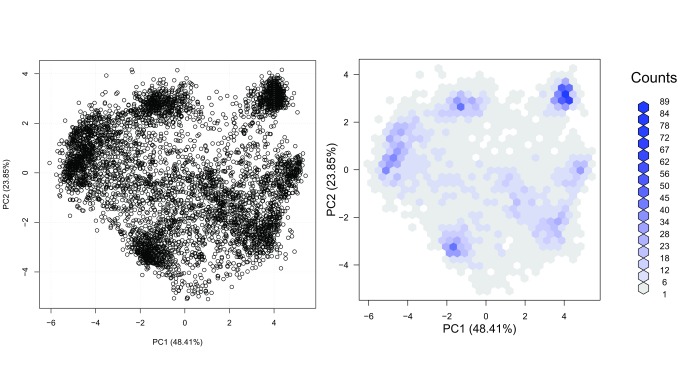
PCA plot of the mouse stem cell data hl. Each dot represents a single protein, and cluster of proteins represent proteins residing in the same sub-cellular niche. The figure on the right bins proteins and represent the bins density to highlight the presence of protein clusters.

In the code chunk below we produce a 2-dimensional PCA plot of the mouse stem cell dataset ( Figure 5). Each point on the plot represents one protein. We can indeed see several distinct protein clusters. We specify fcol = NULL to ignore feature metadata columns and not annotate any feature (protein) with a colour. We will see later how to use this argument to annotate the PCA plot with prior information about sub-cellular localisation.



library ( "pRoloc" )

plot2D (hl, fcol = NULL , col = "black" )

plot2D (hl, method = "hexbin" )
                


In the first instance we advise one to visualise their data without any annotation (i.e. with fcol = NULL), before proceeding with data annotation. The identification of well resolved clusters in the data, constitutes an unbiased assessment of the data structure, demonstrating the successful separation of sub-cellular clusters.

**Figure 6.**
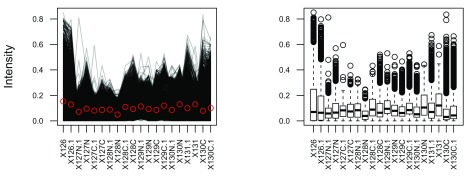
Protein profiles and distribution of channel intensities. The red dots represent the mean relative intensity for each channel.

It is also useful to visualise the relative intensities along the gradient to identify channels displaying particularly low yield. This can be done using the plotDist and boxplot functions, that plot the protein profiles occupancy along the gradient (we also display the mean channel intensities below) and a boxplot of the column intensities. In the two plots displayed on Figure 6, we re-order the TMT channels to pair corresponding channels in the two replicates (rather than ordering the channels by replicate).



par ( mfrow = c (1 ,2 ), ## creates a two-panel figure
	las = 2 ,         ## axis labels orientation
	cex.axis = .7 )   ## axis label size
o <- order (hl$ Iodixonal.Density)

plotDist (hl[, o], pcol = "#00000010" , xlab = "" )

 lines ( colMeans ( exprs (hl[, o])), col = "red" , type = "b" )

boxplot (exprs(hl[, o]))
                


## Markers

In the context of spatial proteomics, a marker protein is defined as a well-known resident of a specific sub-cellular niche in a species *and* condition of interest. Applying this to machine learning (ML), and specifically supervised learning for the task of protein localisation prediction, these markers constitute the labelled training data to use as input to a classification analyses. Defining well-known residents, and obtaining labelled training data for ML analyses can be time consuming, but it is important to define markers that are representative of the multivariate data space and on which a classifier will be trained and generated. *pRoloc* provides a convenience function, addMarkers, to directly add markers to an MSnSet object, as demonstrated in the code chunk below. These marker sets can be accessed using the pRolocmarkers() function. Marker sets are stored as a simple named vector in R, and originate from in-house user-defined sets of markers or from previous published studies ^15^, which are continuosly updated and integrated.



## List available marker sets

pRolocmarkers ()


## 7 marker lists available:
## Arabidopsis thaliana [atha]:
##  Ids: TAIR, 543 markers
## Drosophila melanogaster [dmel]:
##  Ids: Uniprot, 179 markers
## Gallus gallus [ggal]:
##  Ids: IPI, 102 markers
## Homo sapiens [hsap]:
##  Ids: Uniprot, 872 markers
## Mus musculus [mmus]:
##  Ids: Uniprot, 937 markers
## Saccharomyces cerevisiae [scer_sgd]:
##  Ids: SGD, 259 markers
## Saccharomyces cerevisiae [scer_uniprot]:
##  Ids: Uniprot Accession, 259 markers
                


These markers can then be mapped to an MSnSet’s featureNames. The mouse dataset used here has Uniprot IDs stored as the featureNames (see head(featureNames(hl))) and the names of the vector of the mouse markers stored in *pRoloc* ( mmus markers) are also Uniprot IDs (see head(mrk) in the code chunk below, that displays the 6 first markers), so it is straightforward to match names between the markers and the MSnSet instance using the addMarkers function.



## Use mouse markers

mrk <- pRolocmarkers ( species = "mmus" )

head (mrk)


##                 P26039                 Q6PB66                 P11276
##   "Actin cytoskeleton"        "Mitochondrion" "Extracellular matrix"
##                 Q6PR54                 Q05793                 P19096
##  "Nucleus - Chromatin" "Extracellular matrix"              "Cytosol"


## Add mouse markers

hl <- addMarkers (hl, mrk)


## Markers in data:  937 out of 5032


## organelleMarkers
##            40S Ribosome           60S Ribosome      Actin cytoskeleton
##                      27                     43                      13
##                 Cytosol  Endoplasmic reticulum                Endosome
##                      43                     95                      12
##    Extracellular matrix        Golgi apparatus                Lysosome
##                      10                     27                      33
##           Mitochondrion    Nucleus - Chromatin Nucleus - Non-chromatin
##                     383                     64                      85
##              Peroxisome        Plasma membrane              Proteasome
##                      17                     51                      34
##                 unknown
##                    4095
                


We recommend at least 13 markers per sub-cellular class (see the *Optimisation* section for details about the algorithmic motivation of this number). Markers should be chosen to confidently represent the distribution of genuine residents of a sub-cellular niche. We generally recommend a conservative approach in defining markers to avoid false assignments when assigning sub-cellular localisation of proteins of unknown localisation. A more relaxed definition of markers, i.e. one that broadly or over-confidently defines markers, risks the erroneous assignment of proteins to a single location, when, in reality, they reside in multiple locations (including the assumed unique location). One can not expect to identify exact boundaries between sub-cellular classes through marker annotation alone; the definition of these boundaries is better handled algorithmically, i.e. after application of the supervised learning algorithm, using the prediction scores (as described in the *Classification* section, in particular Figure 16).

If the protein naming between the marker sets and the MSnSet dataset are different e.g. the markers are labelled by Uniprot accession numbers and the dataset entries are labelled by Uniprot entry names, one will have to convert and match the proteins according to the appropriate identifier. Sometimes, we find the equivalent entry name, Uniprot ID or accession number is stored in the feature metadata, which makes conversion between identifers relatively straightforward. If this is not the case however, conversion can be performed using *biomaRt*, the Bioconductor annotation resources or any conversion softwares available online.

### Adding user-defined marker lists

It is also possible for users to use their own marker list with the addMarkers function. The user needs to create a named vector of marker localisation, or a create a csv file with two columns (one for the protein names, one for the corresponding sub-cellular marker annotation) and pass the vector or file name respectively to the function. As previously mentioned, the protein names of these markers must match some (but not necessarily all) of the MSnSet’s feature names. See ?addMarkers for more details.

In general, the Gene Ontology (GO) ^17^, and in particular the cellular compartment (CC) namespace are a good starting point for protein annotation and marker definition. It is important to note however that automatic retrieval of sub-cellular localisation information, from *pRoloc* or elsewhere, is only the beginning in defining a marker set for downstream analyses. Expert curation is vital to check that any annotation added is in the correct context for the biological question under investigation.

### Visualising markers

**Figure 7.**
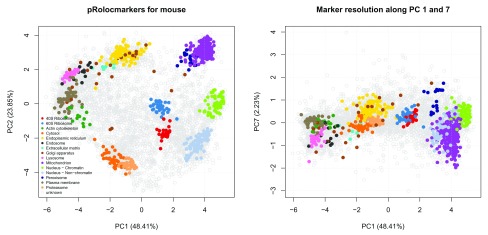
Annotated PCA plots of the hl dataset, as produced with plot2D.

Having added the mouse markers to our fData from the pRolocmarkers, we can now visualise these annotations on the PCA plot using the plot2D function and then use the addLegend function to map the marker classes to the pre-defined colours. As previously mentioned, PCA transforms the original high dimensional data into a set of linearly uncorrelated principal components (PCs) such that the first accounts for as much variability in the data as possible and each succeeding component in turn has the highest variance possible under the constraint that it be orthogonal to the preceding components. We saw in the previous section how visualisation of the PCs is useful for quality control and checking organelle seperation. Adding marker definiton allows one to quickly see if known residents appear in defined clusters. One must be careful though as different organelles may be resolved in different dimensions. For example, we can display the data along the first and seventh PCs using the dims argument. Note that in these calls to the plot2D function, we have omitted the fcol argument and so by default the feature variable named " markers" is used to annotate the plot. We choose to display PCs 1 and 7 to illustrate that while upper principal components explain much less variability in the data (2.23% for PC7, as opposed to 48.41% for PC1), we see that the mitochondrial (purple) and peroxisome (dark blue) clusters can be differentiated, despite the apparent overlap in the visualisation of the two first PCs ( Figure 7).



par ( mfrow = c ( 1 , 2 ))

plot2D (hl, main = "pRolocmarkers for mouse" )

addLegend (hl, cex = .6 )

plot2D (hl, dims = c ( 1 , 7 ), main = "Marker resolution along PC 1 and 7" )
                    


**Figure 8.**
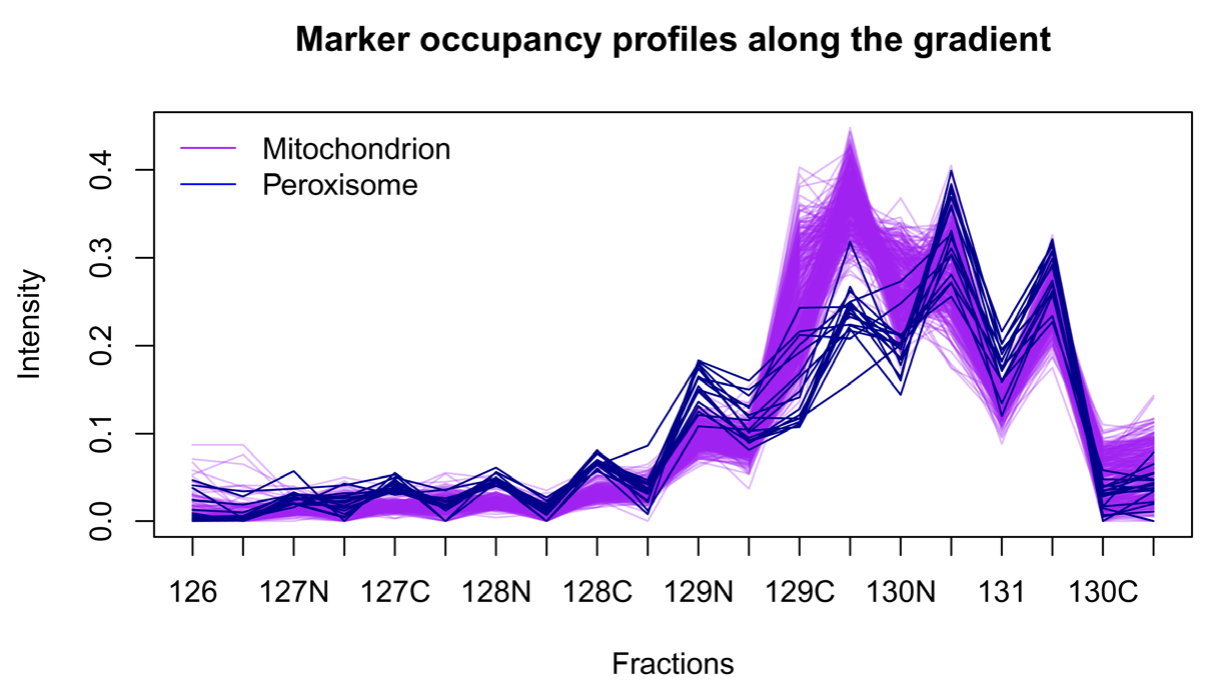
Mitochondrion and peroxisome protein profiles.

This is further highlighted if we plot the profiles of these two clusters using the plotDist function ( Figure 8). The plotDist function is another useful visualisation that relies on marker annotation. It allows one to represent the protein profiles occupancy along the gradient. While the PCA plot enables efficient visualisation of the complete dataset and assessment the relative separation of different sub-cellular niches, comparing profiles of a few marker clusters is useful to assess how exactly they differ (in terms of peak channels, for example). On Figure 8, we plot the profiles of the mitochondrial and peroxisome markers to highlight the differences in the channels labelled with tag 129C, also represented above along the 7th PC on the PCA plot on Figure 7.



hlo <- hl[, order (hl$Iodixonal.Density)]

plotDist (hlo[ fData (hlo)$ markers == "Mitochondrion" , ],
		pcol = "purple" , fractions = "Tag" )

title ( main = "Marker occupancy profiles along the gradient" )

matlines ( t ( exprs (hlo[ fData (hlo)$ markers == "Peroxisome" , ])),
		lty = 1 , col  = "darkblue" , type = "l" )

legend ( "topleft" , c ( "Mitochondrion" , "Peroxisome" ),
        
		lty = 1 , col = c ( "purple" , "blue" ), bty = "n" )
                    


**Figure 9.**
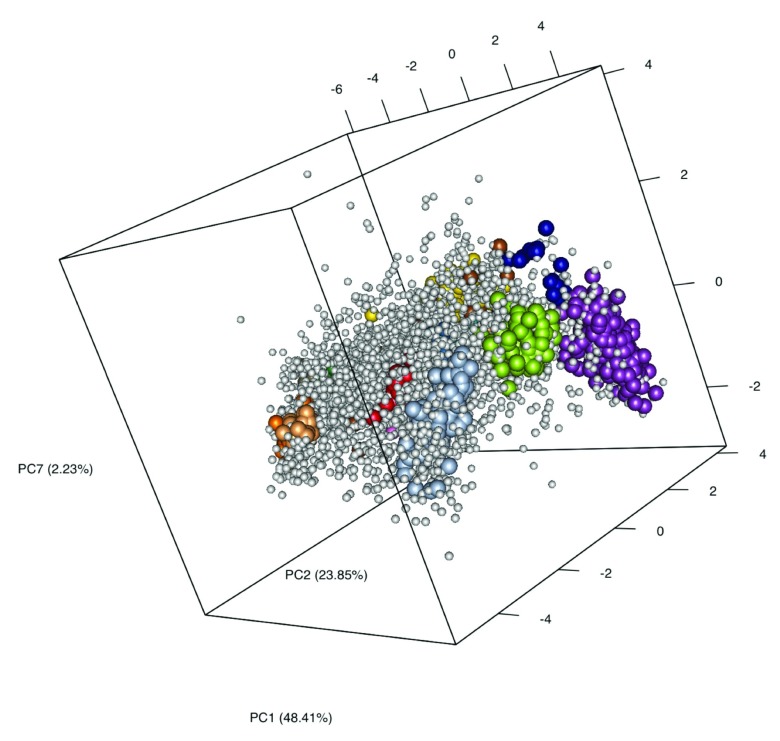
Using the plot3D function to visualise the hl dataset along PCs 1, 2 and 7.

The data can also be visualised along three PCs using the plot3D function ( Figure 9). When produced interactively, the plot can be rotated and zoomed using the mouse.



plotD(h1, dims = c(1, 2, 7))
                    


The default colours for plotting have been defined so as to enable the differentiation of up to 30 classes. If more are provided, different character symbols (circles, squares, ... and empty and solid symbols) are used. The colours and the default plotting characters (solid dots for the markers and empty circles for the features of unknown localisation) can of course be changed, as described in the setStockcol manual page.

As demonstrated in 2 and illustrated in the PCA plot ( Figure 7), the Golgi apparatus proteins (dark brown) display a dynamic pattern, noting sets of Golgi marker proteins that are distributed amongst other subcellular structures, an observation supported by microscopy. As such, we are going to reset the annotation of Golgi markers to unknown using the fDataToUnknown function. It is often used to replace empty strings ( "") or missing values in the markers definition to a common definition of *unknown* localisation.



hl <- fDataToUnknown (hl, from = "Golgi apparatus" , to = "unknown" )

getMarkers (hl)
                    




## organelleMarkers
##            40S Ribosome            60S Ribosome      Actin cytoskeleton
##                      27                      43                      13
##                 Cytosol   Endoplasmic reticulum                Endosome
##                      43                      95                      12
##    Extracellular matrix                Lysosome           Mitochondrion
##                      10                      33                     383
##     Nucleus - Chromatin Nucleus - Non-chromatin              Peroxisome
##                      64                      85                      17
##         Plasma membrane              Proteasome                 unknown
##                      51                      34                    4122
                    


### Features of interest

**Figure 10.**
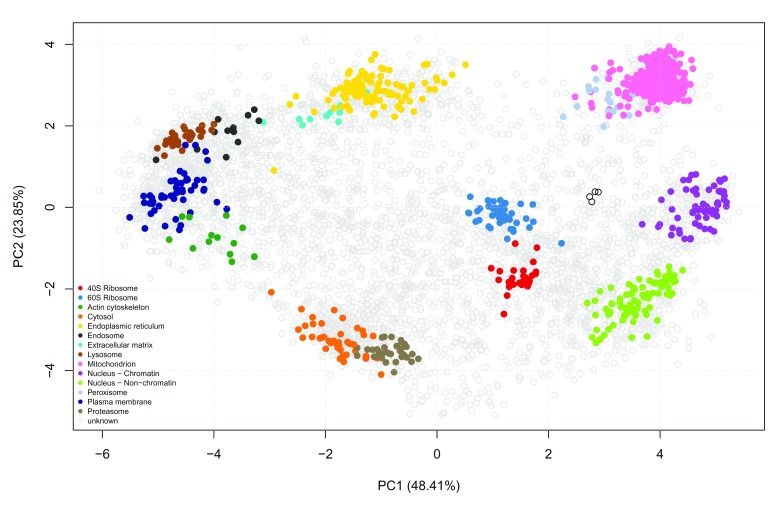
Highlighting protein features of interest.

In addition to adding annotation using the addMarkers function, one can store specific sets of proteins by using the *Features of interest* infrastructure from the *MSnbase* package. If users have specific subsets of proteins they wish to highlight in their data (possibly across multiple experiments) they would first create a FeaturesOfInterest object and then use the highlightOnPlot function to visualise these. For example, if we wanted to highlight proteins with the accession numbers Q8CG48, Q8CG47, Q8K2Z4, and Q8C156, which are some of the proteins known to form part of the 13S condensin complex, we would call the code displayed on Figure 10. Users can also create several sets of FeaturesOfInterest object and store them in a FoICollection.



prots <- c ( "Q8CG48" , "Q8CG47" , "Q8K2Z4" , "Q8C156" )

foi13s <- FeaturesOfInterest ( description = "13S consensin proteins" ,
					fnames= prots,
                        object = hl) 

foi13s
                    




## Traceable object of class "FeaturesOfInterest"
##  Created on Tue May 22 16:07:42 2018
##  Description:
##   13S consensin proteins
##  4 features of interest:
##    Q8CG48, Q8CG47, Q8K2Z4, Q8C156
                    




plot2D (hl)

addLegend (hl, cex = .6 )

highlightOnPlot (hl, foi13s)
                    


It is also worthy of note that it is possible to search for a specific protein of interest by featureNames or using any identifying information found in the fData columns by using the search box on the pRolocVis application part of the *pRolocGUI* package (see section on interactive visualisation). This can be handy for quickly searching and highlighting proteins on the fly, the disavanatge here is that proteins can only be searched for a one-by-one basis.

## Replication

With the aim of maximising the sub-cellular resolution and, consequently, the reliability in protein sub-cellular assignments, we follow the advice in 12 and combine replicated spatial proteomics experiments as described above. Indeed, Trotter *et al*. have shown a significant improvement in protein–organelle association upon direct combination of single experiments, in particular when these resolve different subcellular niches.

Direct comparisons of individual channels in replicated experiments do not provide an adequate, goal-driven assessment of different experiments. Indeed, due to the nature of the experiment and gradient fraction collection, the quantitative channels do not correspond to identical selected fractions along the gradient. For example, in Table 1 below (taken from hl’s pData) TMT channels 127C (among others) in both replicates originate from different sets of gradient fractions (gradient fractions 7–9 and 8–9 for each replicate, respectively). Different sets of gradient fractions are often pooled to obtain enough material and optimise acurate quantitation.

**Table 1.** Differences in gradient fraction pooling.

	Replicate	Tag	Gradient.Fraction	Iodixonal.Density
X127C	1	127C	8 to 9 (pooled)	11.00
X127C.1	2	127C	7 to 9 (pooled)	10.00

**Figure 11.**
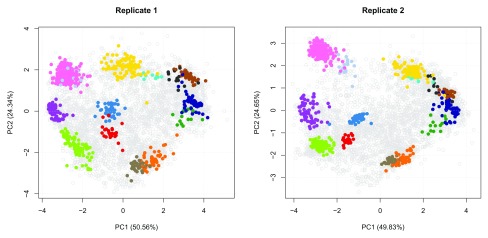
PCA plots of replicates 1 and 2.

The more relevent comparison unit is not a single channel, but rather the complete protein occupancy profiles, which are best visualised experiment-wide on a PCA plot. As such, we prefer to focus on the direct, qualitative comparison of individual replicate PCA plots ( Figure 11), assuring that each displays acceptable sub-cellular resolution. Note that in the code chunk below, we mirror the x-axis to represent the two figures with the same orientation. The interactive "compare" application part of the *pRolocGUI* package is also useful for examining replicate experiments (see the next section *interactive visualisation for details*).



par ( mfrow = c ( 1 , 2 ))

plot2D (hl[, hl$ Replicate == 1], main = "Replicate 1" )

plot2D (hl[, hl$ Replicate == 2], main = "Replicate 2" , mirrorX = TRUE )
                


In addition, the reproducibility can be assessed by performing independent classification analyses on each replicate (see the section on *Supervised machine learning* below) and comparing the the results. Even when the gradient conditions different (for unexpected technical or voluntary reasons, to maximise resolution when combining experiments ^12^), one expects agreement in the most confident organelle assignments.

## Interactive visualisation

Visualisation and data exploration is an important aspect of data analyses allowing one to shed light on data structure and patterns of interest. Using the *pRolocGUI* package, we can interactively visualise, explore and interrogate quantitative spatial proteomics data. The *pRolocGUI* package relies on the shiny framework for reactivity and interactivity. It distributes 3 different GUI’s ( *main* (default), *compare* or *classify*) which are wrapped and launched by the pRolocVis function.

### The main application

**Figure 12.**
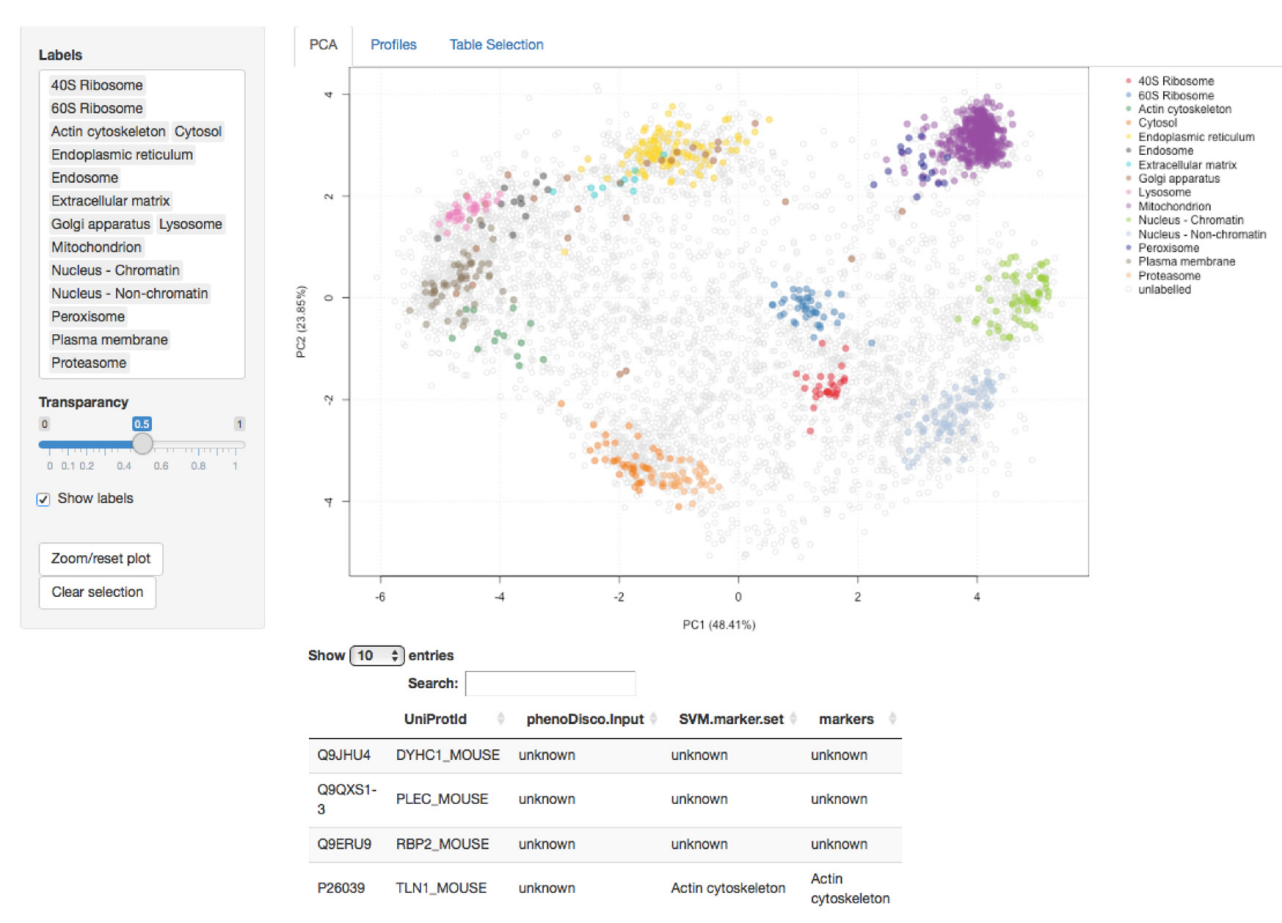
A screen shot of clickable interface and zoomable PCA plot of the main app in the *pRolocGUI* package.

In the below code chunk we lauch the main app ( Figure 12) (note, we do not need to specify the argument, app = "main" as it is the default).



library ( "pRolocGUI" )

pRolocVis (hl)
                    


As diplayed in the screenshot in Figure 12, the *main* application is designed for exploratory data analysis and is divided into 3 tabs: (1) PCA, (2) Profiles and (3) Table selection. The default view upon loading is the *PCA* tab, which features a clickable interface and zoomable PCA plot with an interactive data table for displaying the quantitation information. Particular proteins of interest can be highlighted using the text search box. There is also a *Profiles* tab for visualisation of the protein profiles, which can be used to examine the patterns of proteins of interest. The *Table selection* tab provides an interface to control data table column selection. A short animation https://github.com/lmsimp/bioc-pRoloc-hyperLOPIT-workflow/blob/master/Figures/pRolocVis_pca.gif illustrating the interface is available in the manuscript repository ^18^.

### The compare application

**Figure 13.**
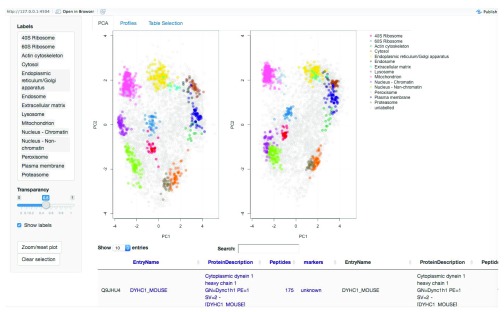
The compare application, main panel.

The *compare* application ( Figure 13) is useful for examining two replicate experiments, or two experiments from different conditions, treatments etc. The compare application is called by default if the input object to pRolocVis is an MSnSetList of 2 MSnSets, but it can also be specified by calling the argument app = "compare". For example, in the code chunk below we first create an MSnSetList of replicates 1 and 2 of the hyperLOPIT data, this is then passed to pRolocVis.



data (hyperLOPIT2015ms3r1)

data (hyperLOPIT2015ms3r2)

hllst <- MSnSetList ( list (hyperLOPIT2015ms3r1, hyperLOPIT2015ms3r2))

pRolocVis (hllst, app = "compare" )
                    


The comparison app loads the two PCA plots side-by-side. Only common proteins between the two data sets are displayed. As per the main application, proteins can be searched, identified and highlighted on both PCA plots and in the dedicated profiles tab. One key feature of the compare application is the ability to re-map the second dataset onto the PCA data space of the first (reference) data set (see ?pRolocVis and the argument remap = TRUE). Using the first dataset as the reference set, PCA is carried out on the first dataset and the standard deviations of the principal components (i.e. the square roots of the eigenvalues of the covariance/correlation matrix) and the matrix of variable loadings (i.e. a matrix whose columns contain the eigenvectors) are stored and then used to calculate the principal components of the second dataset. Both datasets are scaled and centered in the usual way. The first dataset appears on the left, and the second re-mapped data appears on the right. The order of the first (the reference data for remapping) and second dataset can be changed through regeneration/re-ordering of the MSnSetList object.

### The classify application

The final application *classify*, has been designed to view machine learning classification results according to user-specified thresholds for the assignment of proteins to its sub-cellular location, as discussed later in the subsection *Thresholding* in the *Supervised machine learning* section.

## Novelty detection

The extraction of sub-cellular protein clusters can be difficult owing to the limited number of marker proteins that exist in databases and elsewhere. Furthermore, given the vast complexity of the cell, automatic annotation retrieval does not always give a full representation of the true sub-cellular diversity in the data. For downstream analyses, such as supervised machine learning, it is desirable to obtain reliable markers that cover as many sub-cellular niches as possible, as these markers are directly used in the training phase of the ML classification. We find that a lack of sub-cellular diversity in the labelled training data leads to prediction errors, as unlabelled instances can only be assigned to a class that exists in the training data ^19^. In such scenarios, novelty detection can be useful to identify data-specific sub-cellular groupings such as organelles and protein complexes. The phenotype discovery (phenoDisco) algorithm ^19^ is one such method and is available in *pRoloc*. It is an iterative semi-supervised learning method that combines the classification of proteins on existing labelled data with the detection of new clusters.

In addition to extracting new phenotypes, novelty detection methods are also useful for confirming the presence of known or postulated clusters in an unbiased fashion. For example in 2 the phenoDisco algorithm was used to confirm the data-specific presence of the nucleus and nucleus sub-compartments. In the code chunk below, we demonstrate how to do this analysis, highlighting some of the optional arguments and parameters available for phenotype extraction and give some advice on how to interpret the output.

As the phenoDisco algorithm is semi-supervised it uses both labelled (markers) and unlabelled data to explore the data structure and find new sub-cellular data clusters. Thus the first step is to define some input labelled data i.e. markers, that the algorithm will use as input for the supervised learning aspect of the algorithm. As described in 2 we define a set of markers to use as input for the analyses that cover well-known residents from three distinct organelle structures; the mitochondria, plasma membrane and ER, and from three well-known and abundant protein complexes; the proteasome and two ribosomal subunits, 40S and 60S. These input markers are stored in the phenoDisco.Input featureData column of hl and below set by fcol = "phenoDisco.Input". We can use the convenience accessor function getMarkers to print out a table of the markers contained in this marker set. These initial markers were manually curated using information from the UniProt database, the Gene Ontology and the literature.



getMarkers (hl, fcol = "phenoDisco.Input" )


## organelleMarkers
##                          40S Ribosome
##                                    26
##                          60S Ribosome
##                                    43
## Endoplasmic reticulum/Golgi apparatus
##                                    76
##                         Mitochondrion
##                                   261
##                       Plasma membrane
##                                    50
##                            Proteasome
##                                    34
##                               unknown
##                                  4542
                


In the code chunk below we show how to run the phenoDisco function and return a novelty detection result, according to the specified parameters. The algorithm parameters times (number of iterations) and GS (minimum number of proteins required to form a new phenotype) are passed to the function, along with the fcol to tell the algorithm where the input training data is contained.



## As per Christoforou et al (2016),

hl <- phenoDisco (hl, fcol = "phenoDisco.Input" , times = 200 , GS = 60 )
                


The above analysis is computationally intensive and best parallelised over multiple workers. This phenoDisco analysis took 24 hours to complete when parallelised over 40 workers. As such, in the interest of time users can access the above results which are pre-computed and stored along with the pRolocdata package. Please see the Appendix to load these results.

The argument times indicates the number of times we run unsupervied Gaussian Mixture Modelling before defining a new phenotype cluster. The recommended minimum and default value is 100. In the above code chunk we increase the value to times = 200 as we have found for larger datasets (e.g. 5000+ proteins) a higher times is requried for convergence. GS defines the minimum number of proteins allowed per new data cluster and thus heavily influences what type of new clusters are extracted. For example, if a user is interested in the detection of small complexes they may wish to use a small GS = 10, or GS = 20 etc. If they wish to detect larger, more abundant sub-cellular niches a much higher GS would be preferable. Specifying a small GS can be more time consuming than using a larger GS, and there is a trade off between finding interesting small complexes and those that may not be of interest as we find there is a tendancy to find more noise when using a small GS compared to using a higher one.

One may also consider increasing the search space for new data clusters by increasing the value of the parameter G. This defines the number of GMM components to test and fit; the default is G = 1:9 (the default value in the *mclust* package ^20^). One should note that the decreasing the GS, and increasing the values of the arguments times, G (among other function arguments, see ?phenoDisco) will heavily influence (increase) the total time taken to run the algorithm. phenoDisco supports parallelisation and we strongly suggest you make use of a parallel processing to run these analyses.

The ouput of running the phenoDisco algorithm is an MSnSet containing the new data clusters, appended to the featureData under the name pd. The results can be displayed by using the getMarkers function. We see that 5 new phenotype data clusters were found.



hl


## MSnSet (storageMode: lockedEnvironment)
## assayData: 5032 features, 20 samples
##   element names: exprs
## protocolData: none
## phenoData
##   sampleNames: X126 X127N ... X131.1 (20 total)
##   varLabels: Replicate Tag Gradient.Fraction Iodixonal.Density
##   varMetadata: labelDescription
## featureData
##   featureNames: Q9JHU4 Q9QXS1-3 ... Q9Z2R6 (5032 total)
##   fvarLabels: uniprot.accession uniprot.id ... pd (27 total)
##   fvarMetadata: labelDescription
## experimentData: use 'experimentData(object)'
## Annotation:
## - - - Processing information - - -
## Added markers from  'mrk' marker vector. Tue May 22 16:07:41 2018
## Added markers from  'pdres' marker vector. Tue May 22 16:07:42 2018
##  MSnbase version: 2.7.1


getMarkers (hl, fcol = "pd" )


## organelleMarkers
##                          40S Ribosome
##                                   106
##                          60S Ribosome
##                                    95
## Endoplasmic reticulum/Golgi apparatus
##                                   393
##                         Mitochondrion
##                                   525
##                           Phenotype 1
##                                   300
##                           Phenotype 2
##                                   253
##                           Phenotype 3
##                                   203
##                           Phenotype 4
##                                    74
##                           Phenotype 5
##                                    91
##                       Plasma membrane
##                                   421
##                            Proteasome
##                                    92
##                               unknown
##                                  2479
                


**Figure 14.**
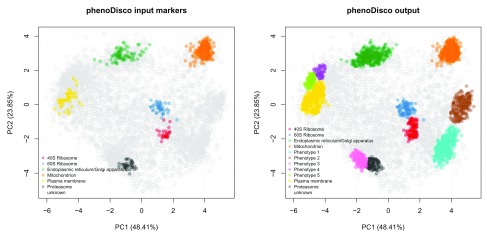
Results of the novelty detection algorithm.

We can plot the results using the plot2D function ( Figure 14).



## Re-order the colours for the phenoDisco output

cl <- getMarkerClasses (hl, "pd" )

cols <- getStockcol ()[ seq (cl)]

ind <- grep ( "Pheno" , cl, invert = TRUE )
cols[ind] <- getStockcol ()[ seq (cl)][ 1 : length (ind)]
cols[-ind] <- getStockcol ()[ seq (cl)][( length (ind) + 1 ): length (cl)]


## Plot the input and output
par ( mfrow = c ( 1 , 2 ))

plot2D (hl, fcol = "phenoDisco.Input" ,
        main = "phenoDisco input markers" , col = getStockcol ()[ 1: 6 ])

addLegend (hl, fcol = "phenoDisco.Input" , cex = .7 )

plot2D (hl, fcol = "pd" , main = "phenoDisco output" , col = cols)

addLegend (hl, fcol = "pd" , cex = .7 , col = cols)
                


The five new phenotype data clusters can be extracted and examined. In the code chunk below we write the results to a .csv file using the write.exprs function. We use the argument fDataCols to specify which columns of the featureData to write.



fData (hl)$ pd <- as.character ( fData (hl)$ pd)

write.exprs (hl, fcol = "pd" , file = "pd-results.csv" , sep = "," )
                


We can also examine each phenotype interactively and visualise their protein profiles by using the pRolocVis function in the *pRolocGUI* package. We found that phenotype 1 was enriched in nucleus associated proteins, phenotype 2 in chromatin associated proteins, phenotype 3 in cytosolic and phenotypes 4 and 5 in both lysosomal and endosomal proteins.



pRolocVis (hl, fcol = "pd" )
                


## Supervised machine learning

Supervised machine learning, also known as classification, is an essential tool for the assignment of proteins to distinct sub-cellular niches. Using a set of labelled training examples i.e. markers, we can train a machine learning classifier to learn a mapping between the data i.e. the quantitative protein profiles, and a known localisation. The trained classifier can then be used to predict the localisation of a protein of unknown localisation, based on its observed protein profiles. To date, this method has been extensively used in spatial quantitative proteomics to assign thousands of proteins to distinct sub-cellular niches ^2, 12, 21– 24^.

There are several classification algorithms readily available in *pRoloc*, which are documented in the dedicated *pRoloc* machine learning techniques vignette. We find the general tendency to be that it is not the choice of classifier, but the improper optimisation of the algorithmic parameters, that limits classification accuracy. Before employing any classification algorithm and generating a model on the training data, one must first find the optimal parameters for the algorithm of choice.

### Optimisation

In the code chunk below we use a Support Vector Machine (SVM) to learn a classifier on the labelled training data. As previously mentioned, one first needs to train the classifier’s parameters before an algorithm can be used to predict the class labels of the proteins with unknown location. One of the most common ways to optimise the parameters of a classifier is to partition the labelled data into training and testing subsets. In this framework parameters are tested via a grid search using cross-validation on the training partition. The best parameters chosen from the cross-validation stage are then used to build a classifier to predict the class labels of the protein profiles on the test partition. Observed and expected classication results can be compared, and then used to assess how well a given model works by getting an estimate of the classifiers ability to achieve a good generalisation i.e. that is given an unknown example predict its class label with high accuracy. In *pRoloc*, algorithmic performance is estimated using stratified 80/20 partitioning for the training/testing subsets respectively, in conjuction with five-fold cross-validation in order to optimise the free parameters via a grid search. This procedure is usually repeated 100 times and then the best parameter(s) are selected upon investigation of classifier accuracy. We recommend a minimum of 13 markers per sub-cellular class for stratified 80/20 partitioning and 5-fold cross-validation; this allows a minimum of 10 examples for parameter optimisation on the training partition i.e. 2 per fold for 5-fold cross-validation, and then 3 for testing the best parameters on the validation set.

Classifier accuracy is estimated using the macro F1 score, i.e. the harmonic mean of precision and recall. In the code chunk below we demonstrate how to optimise the free parameters, sigma (the inverse kernel width for the radial basis kernel) and cost (the cost of constraints violation), of a classical SVM classifier with a Gaussian kernel using the function svmOptimisation. As the number of labelled instances per class varies from organelle to organelle, we can account for class imbalance by setting specific class weights when generating the SVM model. Below the weights, w are set to be inversely proportional to the class frequencies.



w <- table ( getMarkers (hl, verbose = TRUE ))


## organelleMarkers
##            40S Ribosome            60S Ribosome   Actin cytoskeleton
##                      27                      43                   13
##                 Cytosol   Endoplasmic reticulum             Endosome
##                      43                      95                   12
##    Extracellular matrix                Lysosome        Mitochondrion
##                      10                      33                  383
##     Nucleus - Chromatin Nucleus - Non-chromatin           Peroxisome
##                      64                      85                   17
##         Plasma membrane              Proteasome              unknown
##                      51                      34                 4122


w <- 1/ w[ names (w) != "unknown" ]
                    


In the code chunk below we then pass these weights to the svmOptimisation function. Once again, we provide the optimisation results for users to load directly if they wish to save computational time (see the Appendix for details).



## 100 rounds of optimisation with five-fold cross-validation

params <- svmOptimisation (hl, fcol = "markers" ,
						times = 100 , xval = 5 ,
						class.weights = w)
                    


As mentioned previously, we rely on the default feature variable "markers" to define the class labels and hence do not need to specify it in the above code chunk. To use another feature variables, one needs to explicitly specify its name using the fcol argument (for example fcol = "markers2").

The output params is an object of class GenRegRes; a dedicated container for the storage of the design and results from a machine learning optimisation. To assess classifier performance we can examine the macro F1 scores and the most frequently chosen parameters. A high macro F1 score indicates that the marker proteins in the test dataset are consistently and correctly assigned by the the algorithm. Often more than one parameter or set of parameters gives rise to the best generalisation accuracy. As such it is always important to investigate the model parameters and critically assess the best choice. The f1Count function counts the number of parameter occurences above a certain F1 value. The best choice may not be as simple as the parameter set that gives rise to the highest macro F1 score. One must be careful to avoid overfitting, and choose parameters that frequently provide high classification accuracy. Below, we see that only a sigma of 0.1 produces macro F1 scores above 0.6, but that a cost of 16 is much more frequently chosen than lower values.



f1Count (params, 0.6 )


##     4  8 16
## 0.1 1 10 89
                    


**Figure 15.**
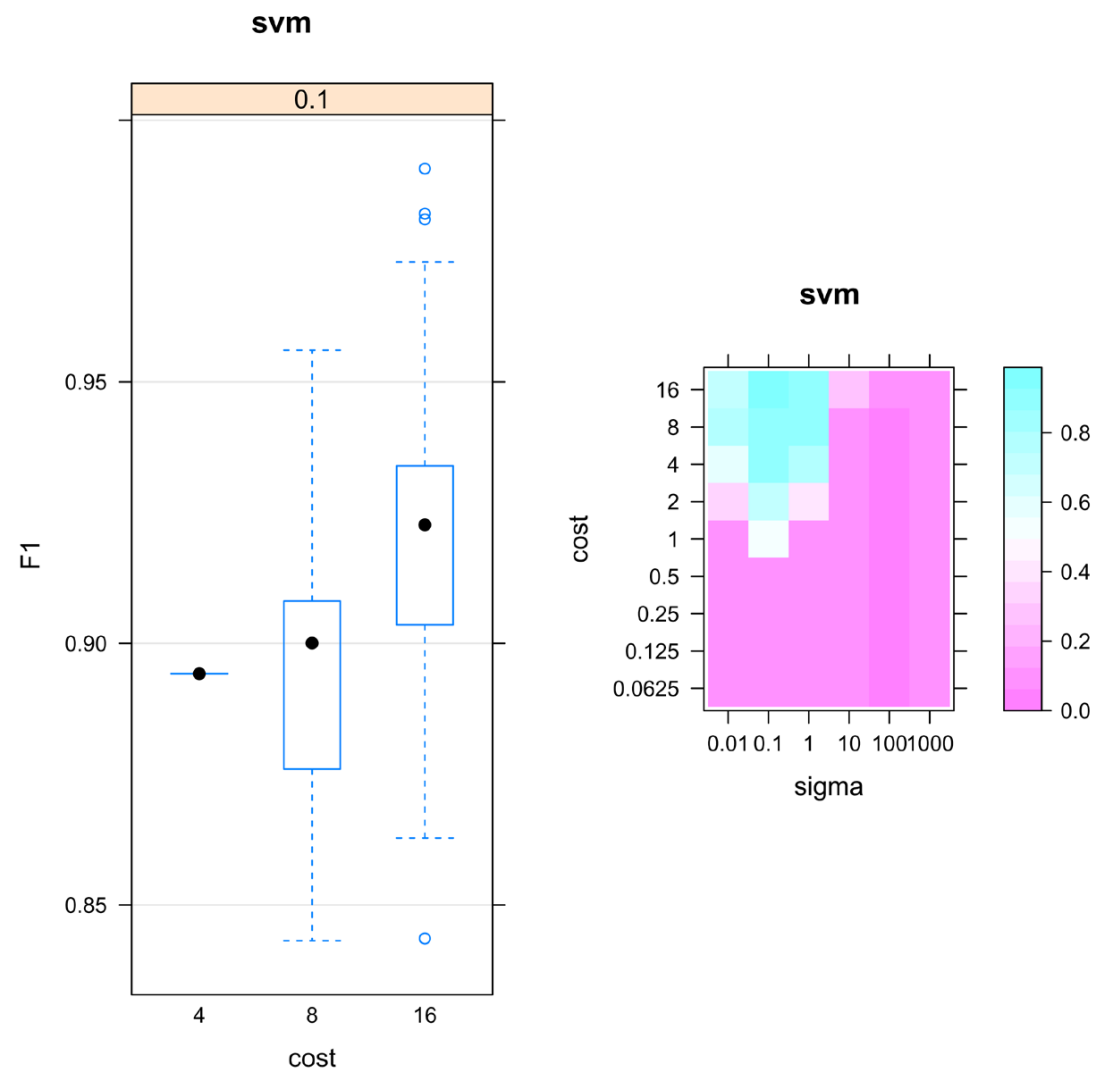
Assessment of the classification model parameter optimisation.

The parameter optimistion results can also be visualised as a boxplot or heatmap, as shown in Figure 15. The plot method for GenRegRes object shows the respective distributions of the 100 macro F1 scores for the best cost/sigma parameter pairs, and levelPlot shows the averaged macro F1 scores, for the full range of parameter values. These figures also indicate that values of 0.1 and 16 for sigma and cost consistently deliver better classification scores.



plot (params)

levelPlot (params)
                    


By using the function getParams we can extract the best set of parameters. Currently, getParams retrieves the first best set automatically, but users are encouraged to critically assess whether this is the most wise choice (which it is, as demonstrated above).



(best <- getParams (params))


## sigma  cost
##   0.1  16.0
                    


Once we have selected the best parameters we can then use them to build a classifier from the labelled marker proteins.

### Classification

We can use the function svmClassification to return a classification result for all unlabelled instances in the dataset corresponding to their most likely sub-cellular location. The algorithm parameters are passed to the function, along with the class weights. As above, the fcol argument does not need to be specified as we use the labels defined in the default "markers" feature variable.



hl <- svmClassification (hl, params, class.weights = w, fcol = "markers" )
                    


In the code chunk above, we pass the whole params parameter results and, internally, the first pair that return the highest F1 score are returned (using the getParams function above). It is advised to always check that these are actually good parameters and, if necessary, set them explicitly, as shown below.



hl <- svmClassification (hl, cost = 16 , sigma = 0.1 , class.weights = w, fcol = "markers" )
                    


Automatically, the output of the above classification, the organelle predictions and assignment scores, are stored in the featureData slot of the MSnSet. In this case, they are given the labels svm and svm.scores for the predictions and scores respectively. The resultant predictions can be visualised using plot2D. In the code chunk below plot2D is called to generate a PCA plot of the data and fcol is used to specify where the new assignments are located e.g. fcol = "svm".

**Figure 16.**
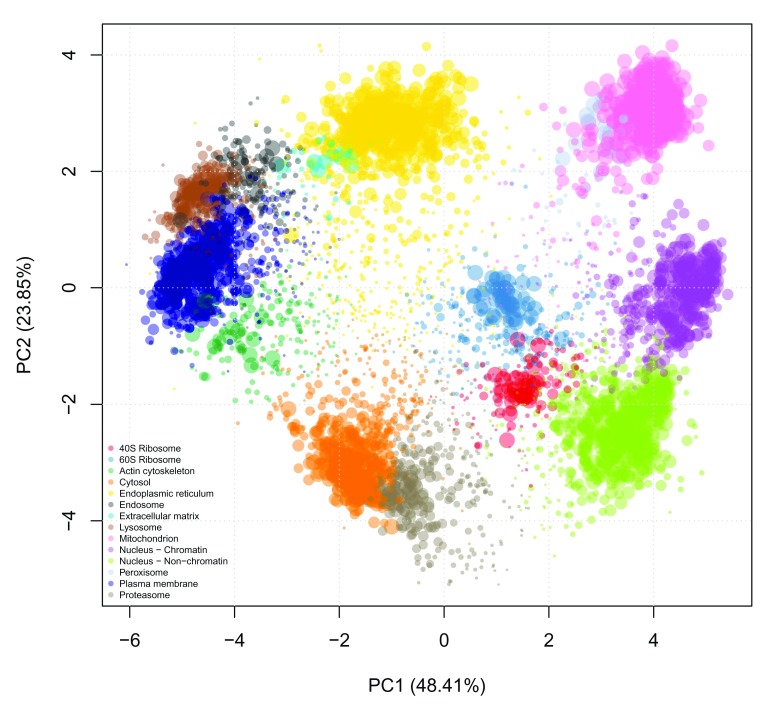
Classification results. Colours indicate class membership and point size are representative of the classification confidence.

Additionally, when calling plot2D we can use the cex argument to change the size of each point on the plot to be proportional to the SVM score ( Figure 16). This gives an initial overview of the high scoring localisations from the SVM predictions.



## set point size of each protein to be proportional to the svm score

ptsze <- exp ( fData (hl)$ svm.scores) - 1

## plot new predictions

 plot2D (hl, fcol = "svm" , cex = ptsze)

addLegend (hl, fcol = "svm" , where = "bottomleft" , bty = "n" , cex = .5 )
                    


The adjustment of the point size intuitively confers important information that is more difficult to define formally (that we will address in the next section). The classifier (SVM in our case, but this is also valid of other classifiers) defines boundaries based on the labelled marker proteins. These class/organelle boundaries define how non-assigned proteins are classified and with what confidence.

### Thresholding

It is common when applying a supervised classification algorithm to set a specific score cutoff on which to define new assignments, below which classifications are kept unknown/unassigned. This is important as in a supervised learning setup, proteins can only be predicted to be localised to one of the sub-cellular niches that appear in the labelled training data. We can not guarantee (and do not expect) that the whole sub-cellular diversity is represented in the labelled training data as (1) finding markers that represent the whole diversity of the cell is challenging (especially obtaining dual- and multiply-localised protein markers) and (2) many sub-cellular niches contain too few proteins to train on (see above for a motivation of a minimum of 13 markers).

**Figure 17.**
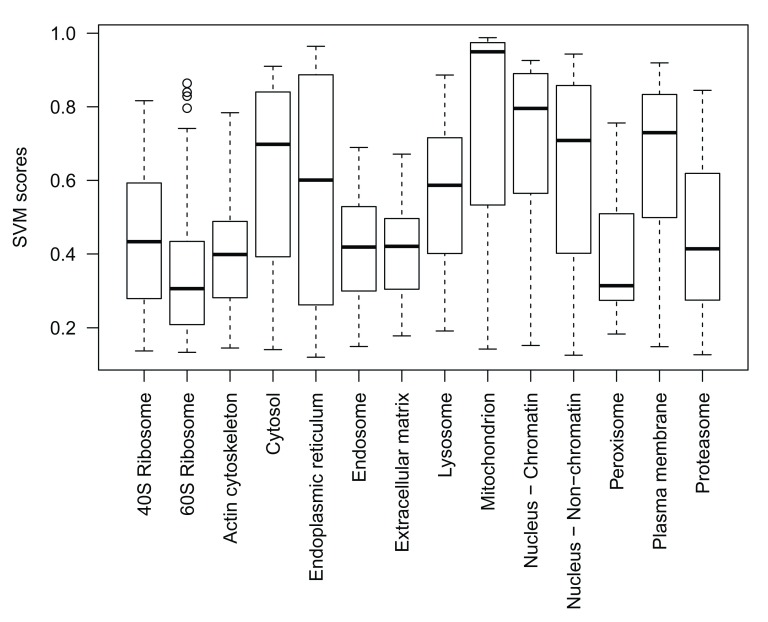
Visualistion of class-specific classification score distribution.

Deciding on a threshold is not trivial as classifier scores are heavily dependent upon the classifier used and different sub-cellular niches can exhibit different score distributions, as highlighted in the boxplot below. We recommend users to set class-specific thresholds. In the code chunk below we display a boxplot of the score distributions per organelle ( Figure 17).



 ## First remove the markers

preds <- unknownMSnSet (hl)

## Plot a boxplot of the scores of each organelle

par ( oma = c ( 10.5 , 0 , 0 , 0 )) ## sets outer margins

boxplot (svm.scores ~ svm, data = fData (preds),
        ylab = "SVM scores" , las = 2 )
                    


There are many ways to set thresholds and the choice of method will depend on the biological question and experimental design at hand. One viable approach in the frame of the above experimetal design would be to manually set a FDR, say 5%, per organelle. To do this the user would examine the top scoring predictions for each organelle, and then set a threshold at the score at which they achieve 5% of false assignments per organelle. The definintion of a false assignment would depend on the information available, for example, validity or lack of validity for the localisation from another experiment as reported in the literature or a reliable database. If such information is not available, one crude method is to set a threshold per organelle by extracting the median or 3rd quartile score per organelle. For example, in the code chunk below, we use the orgQuants function to extract the median organelle scores and then pass these scores to the getPredictions function to extract the new localisations that meet this scoring criteria. Any sub-cellular predictions that fall below the specified thresholds are labelled as unknown.



(ts <- orgQuants (hl, fcol = "svm" , scol = "svm.scores" , mcol = "markers" , t = .5 ))
					




                        ##            40S Ribosome            60S Ribosome     Actin cytoskeleton
##               0.4343633               0.3047074          	0.3832276
##                 Cytosol   Endoplasmic reticulum 	 	 Endosome
##               0.6910080               0.6085463              0.4233563
##    Extracellular matrix                Lysosome          Mitochondrion
##               0.4220800               0.5889173              0.9497770
##     Nucleus - Chromatin Nucleus - Non-chromatin             Peroxisome
##               0.7936060               0.7103170              0.3143802
##         Plasma membrane              Proteasome
##               0.7159213               0.4144325
##            40S Ribosome            60S Ribosome     Actin cytoskeleton
##               0.4343633               0.3047074              0.3832276
##                 Cytosol   Endoplasmic reticulum               Endosome
##               0.6910080               0.6085463              0.4233563
##    Extracellular matrix                Lysosome          Mitochondrion
##               0.4220800               0.5889173              0.9497770
##     Nucleus - Chromatin Nucleus - Non-chromatin             Peroxisome
##               0.7936060               0.7103170              0.3143802
##         Plasma membrane              Proteasome
##               0.7159213               0.4144325
                    




hl <- getPredictions (hl, fcol = "svm" , scol = "svm.scores" , mcol = "markers" , t = ts )
                    




                        ## ans
##            40S Ribosome            60S Ribosome     Actin cytoskeleton
##                      84                     172                     85
##                 Cytosol   Endoplasmic reticulum               Endosome
##                     296                     476                    103
##    Extracellular matrix                Lysosome          Mitochondrion
##                      25                     124                    522
##     Nucleus - Chromatin Nucleus - Non-chromatin             Peroxisome
##                     230                     342                     38
##         Plasma membrane              Proteasome                unknown
##                     320                     157                   2058
                    


The organelle threshold ( ts above) can also be set manually using an interactive app (see below) or by using a named vector of thresholds, as shown in the putative example below for 4 organelles.



(ts <- setNames ( c ( 0.612 , 0.701 , 0.81 , 0.92 ), c ( "PM" , "Mito" , "Golgi" , "ER" )))


##    PM  Mito Golgi    ER
## 0.612 0.701 0.810 0.920
                    


**Figure 18.**
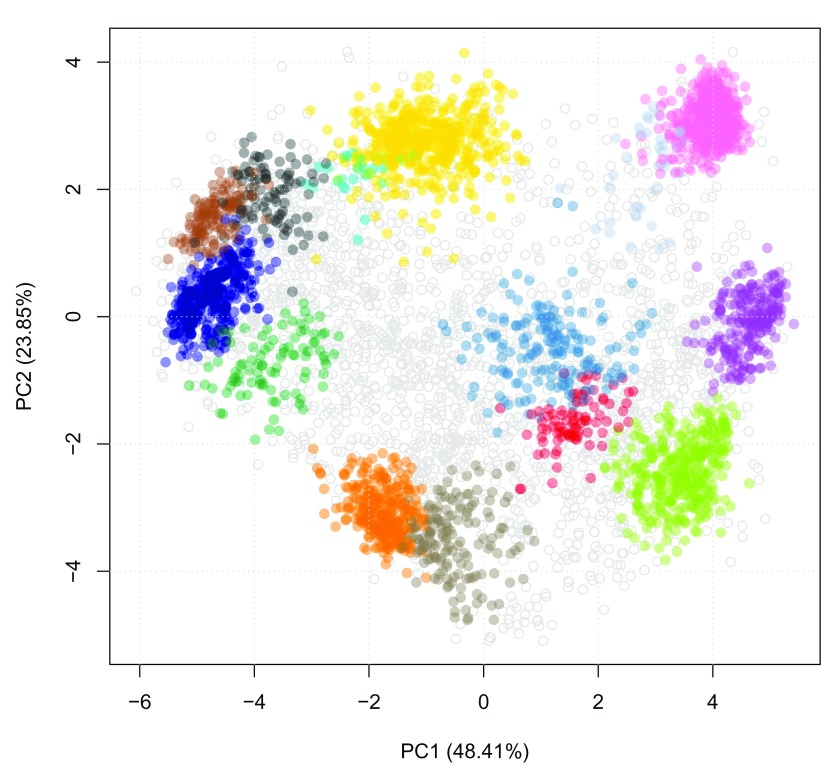
Results of the localisation preductions after thresholding.



The output of getPredictons is the original MSnSet dataset with a new feature variable appended to the feature data called fcol.pred (i.e. in our case svm.pred) containing the prediction results. The results can also be visualised using plot2D function ( Figure 18) and extracted by retrieving that specific column from the feature metadata using, for example, fData(hl)$svm.pred.



plot2D (hl, fcol = "svm.pred" )
                    


library ( "pRolocGUI" )

pRolocVis (hl, app = "classify" , fcol = "svm" , scol = "svm.scores" , mcol = "markers" )
                    


**Figure 19.**
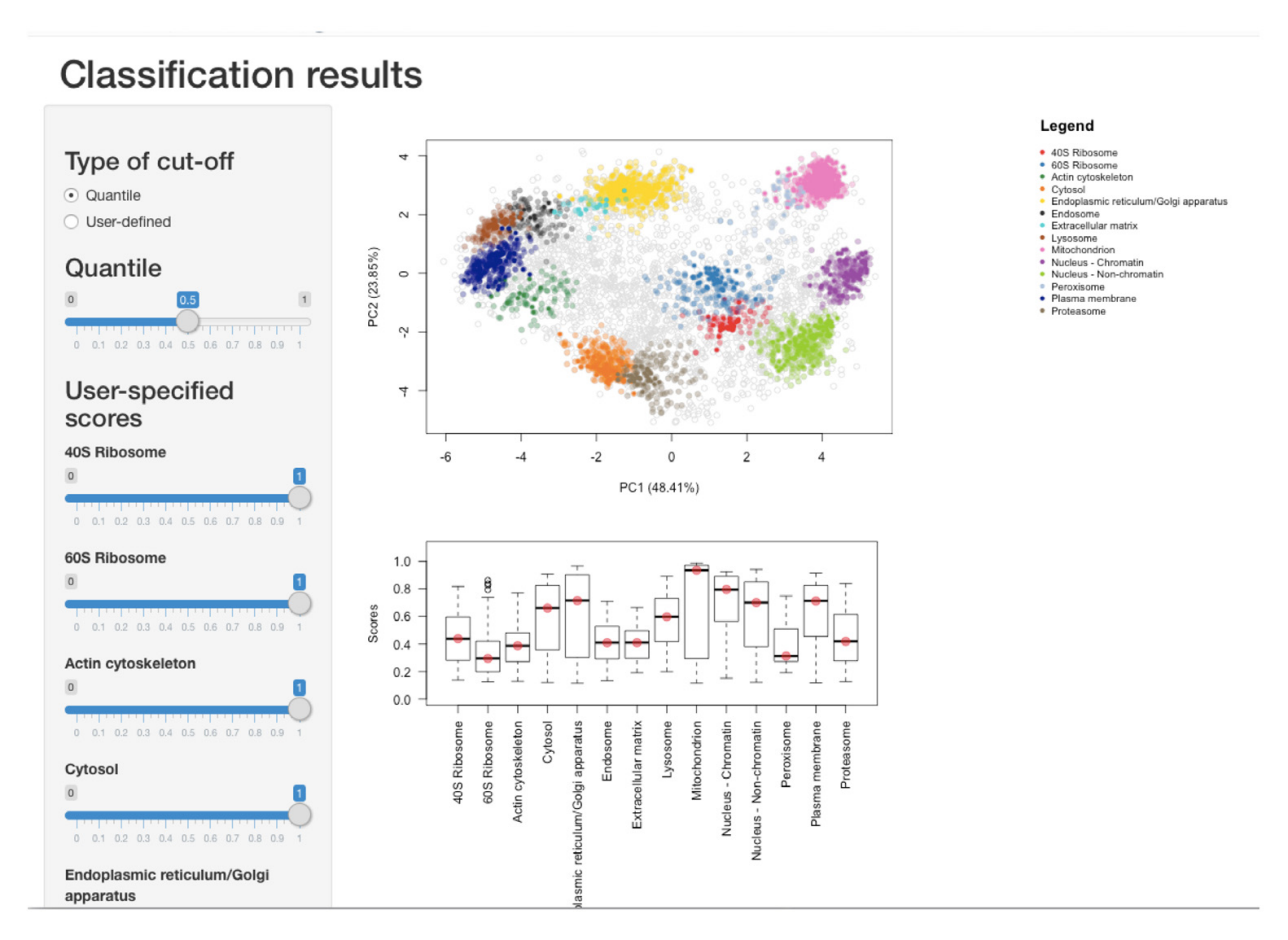
The classify application enable the interactive exploration of classification score thresholding.

There is also a dedicated interactive application to help users examine these distributions in the *pRolocGUI* package ( Figure 19). This app can be launched via the pRolocVis function and specifying the argument app = "classify" along with the relevent fcol, scol and mcol which refer to the columns in the feature data that contain the new assignments, assignment scores and markers respectively (see also fvarLabels(svmres)).

The data is loaded and displayed on a PCA plot and a boxplot is used to display the classifier scores by data class. On the left, there is a sidebar panel with sliders to control the thresholds upon which classifications are made. There are two types of cut-off that the user can choose from: (1) *Quantile* and (2) *User-defined*. By default, when the application is launched quantile scoring is selected and set to 0.5, the median. The class-specific score thresholds that correspond to selecting the desired quantile are shown as red dots on the boxplot. The assignments on the PCA plot are also updated according to the selected threshold. The quantile threshold can be set by moving the corresponding quantile slider. If the users wishes to set their own cut-offs, the *User-defined* radio button must be selected and then the sliders for defining user-specified scores become active and the scores are highlighted on the boxplot by blue dots. For more information we refer users to the *pRolocGUI* tutorial vignette.

## Transfer learning

In addition to high quality MS-based quantitative proteomics data, there exist a number of other sources of information that are freely available in the public domain that may be useful to assign a protein to its sub-cellular niche. For example, imaging from immunofluorescence microscopy, protein annotations and sequences, and protein-protein interactions among others, represent a rich and vast source of complementary information. We can integrate this auxiliary information with our primary MS-based quantitative data using a paradigm known as transfer learning (TL). The integration of data between different technologies is one of the biggest challenges in computational biology to date and the *pRoloc* package provides functionality to do such analyses. We recently developed two transfer learning algorithms using a *k*-NN and SVM framework and applied them to the task of protein localisation prediction ^25^. In this section we will begin with explaining the concept of transfer learning and then show how to apply this in the frame of spatial proteomics and protein localisation prediction.

In TL one typically has a primary task that one wishes to solve, and some complementary (often heterogeneous) auxiliary information that is related to the primary learning objective, that can be used to help solve the primary goal. For example, here our primary task is to assign proteins to their sub-cellular niche with high generalisation accuracy from data collected from quantitative MS-based experiments. We have already seen that straightforward supervised ML works well for these types of experiments, however, Transfer learning is particularly useful for classes that are not as well separated.

In the example below we extract Gene Ontology (GO) information to use as an auxiliary data source to help solve our task of protein localisation prediction.

Using the functions setAnnotationParams and makeGoSet we can contruct an auxiliary MSnSet of GO terms, from the primary data’s features i.e. the protein accession numbers. All the GO terms associated to each accession number are retrieved and used to create a binary matrix where a 1 (0) at position ( *i*, *j*) indicates that term *j* has (not) been used to annotate protein *i*. The GO terms are retrieved from an appropriate repository using the *biomaRt* package. The specific Biomart repository and query will depend on the species under study and the type of identifiers. The first step is to construct the annotation parameters that will enable to perform the query, which is done using setAnnotationParams. Typing into the R console par <- setAnnotationParams() will present two menus, firstly asking you to identify the species of study, and then what type of identifier you have used to annotate the proteins in your MSnSet. It is also possible to pass arbitrary text to match the species e.g. in the code chunk below we pass "Mus musculus", and the identifier type for our data (see featureNames(hl)) which is "Uniprot/Swissprot", for the Biomart query.



par <- setAnnotationParams ( inputs = c ( "Mus musculus" , "UniProt/Swissprot" ))
                


Now we have contructed the query parameters we can use the makeGoSet function to retrieve and build an auxiliary GO MSnSet as described above. By default, the cellular component terms are downloaded, without any filtering on evidence codes. It is also possible to download terms from the molecular function and biological process GO namespaces, and also apply filtering based on evidence codes as desired, see ?makeGoSet for more details. (We also provide the pre-computed gocc object for users if they wish to load directly, please see the Appendix).



gocc <- makeGoSet (hl, params = par, namespace = "cellular_component" )
                


The function makeGoSet uses the *biomaRt* package to query the relevent database (e.g. Ensembl, Uniprot) for GO terms. All GO terms that have been observed for the 5032 proteins in the hyperLOPIT dataset are retrieved. Users should note that the number of GO terms used is also dependent on the database version queried and thus is always subject to change. We find it is common to see GO terms with only one protein assigned to that term. Such terms do not bring any information for building the classifier and are thus removed using the filterBinMSnSet function.



gocc <- filterBinMSnSet (hl)
                


Now that we have generated our auxiliary data, we can use the *k*-NN implementation of transfer learning available in *pRoloc* to integrate this with our primary MS-based quantitative proteomics data using the functions knntlOptimisation to estimate the free-parameters for the integration, and knntlClassification to do the predictions. We have shown that using transfer learning results in the assignment of proteins to sub-cellular niches with a higher generalisation accuracy than using standard supervised machine learning with a single source of information ^25^.

### TL optimisation

The first step, as with any machine learning algorithm, is to optimise any free paramaters of the classifier. For the *k*-NN TL classifier there are two sets of parameters that need optimising: the first set are the *k*’s for the primary and auxiliary data sources required for the nearest neighbour calculations for each data source. The second set of parameters (noted by a vector of *θ* weights) that require optimising are the class weights, one per subcellular niche, that control the proportion of primary and auxiliary data to use for learning. A weight can take any real value number between 0 and 1. A weight of *θ* = 1 indicates that all weight is given to the primary data (and this implicitly implies that a weight of 1 *− θ* = 0 is given to the auxiliary data), and similarly a weight of *θ* = 0 implies that all weight is given to the auxiliary data (so 0 is given to the primary source). If we conduct a parameter search and test weights *θ* = 0, 1 */*3, 2 */*3, 1 for each class, and if we have, for example 10 subcellular niches, this will result in 4 ^10^ different combinations of parameters to test. The parameter optimisation is therefore time consuming and as such we recommend making use of a computing cluster (code and submissing scripts are also available in the supporting information). The markers in the hl dataset contain 14 subcellular classes. If we examine these markers and classes on the PCA plot above we can see that in particular the two ribosomes and two nuclear compartments are highly separated along the first two components, this is also evident from the profiles plot which gives us a good indication that these subcellular niches are well-resolved in the hyperLOPIT dataset. Transfer learning is particularly useful for classes that are not as well separated. We find that subcellular niches that are well-separated under hyperLOPIT and LOPIT obtain a class score of 1 (i.e. use only primary data from transfer learning ^25^). Therefore, for the optimisation stage of the analyses we can already infer a subcellular class weight of 1 for these niches and only optimise over the remaining organelles. This can significantly cut down optimisation time as by removing these 4 classes from the optimisation (and not the classification) we only have 4 ^10^ class weight combinations to consider instead of 4 ^14^ combinations.

In the example below we first remove these 4 classes from the marker set and create a new marker set called tlmarkers. Then we re-run the knnOptimisation for each data source and then run the knntlOptimisation with the 10 remaining classes. (Note: this is not run live as the hl dataset with 10 classes, 707 markers and 4 ^10^ combinations of parameters takes around 76 hours to run on the University of Cambridge HPC using 256 workers. To save time for users, the results of the following optimisation are pre-computed and provided with the pRolocdata package. Please see the Appendix for details on how to load these directly.



## create new markers column for tl markers

fData (hl)$ tlmarkers <- fData (hl)$ markers

fData (gocc)$ tlmarkers <- fData (gocc)$ markers


## Remove 4 classes

torm <- c ( "40S Ribosome" , "60S Ribosome" ,
            
            "Nucleus - Chromatin" ,
            
            "Nucleus - Non-chromatin" )

for (i in seq (torm)) {
  
	hl <- fDataToUnknown (hl, from = torm[i], fcol = "tlmarkers" )
  
    gocc <- fDataToUnknown (gocc, from = torm[i], fcol = "tlmarkers" )
}

getMarkerClasses (hl, fcol = "tlmarkers" )


##  [1] "Actin cytoskeleton"    "Cytosol"
##  [3] "Endoplasmic reticulum" "Endosome"
##  [5] "Extracellular matrix"  "Lysosome"
##  [7] "Mitochondrion"         "Peroxisome"
##  [9] "Plasma membrane"       "Proteasome"


getMarkerClasses (gocc, fcol = "tlmarkers" )


##  [1] "Actin cytoskeleton"    "Cytosol"
##  [3] "Endoplasmic reticulum" "Endosome"
##  [5] "Extracellular matrix"  "Lysosome"
##  [7] "Mitochondrion"         "Peroxisome"
##  [9] "Plasma membrane"       "Proteasome"
                    


**TL optimisation stage 1** Run knnOptimisation to get the best *k*’s for each data source.



## get best k's

kpopt <- knnOptimisation (hl, fcol = "tlmarkers" )

kaopt <- knnOptimisation (gocc, fcol = "tlmarkers" )
                    


From examining the parameter search plots as described in section *Optimisation*, we find the best *k*’s for both the primary and auxiliary are 3.

**TL optimisation stage 2** Run knntlOptimisation to get the best transfer learning weights for each sub-cellular class.



## Set appropriate parallelisation backend and
## number of workers for the tl

par <- SnowParam ( 255L , type = "MPI" )
                    




## Now peform tl optimisation

tlopt <- knntlOptimisation (hl, gocc, fcol = "tlmarkers" ,
                              
					length.out = 4 , times = 50 ,
                              
					xval = 5 , k = c ( 3 , 3 ),
                              
						BPPARAM = par)
                    


**Figure 20.**
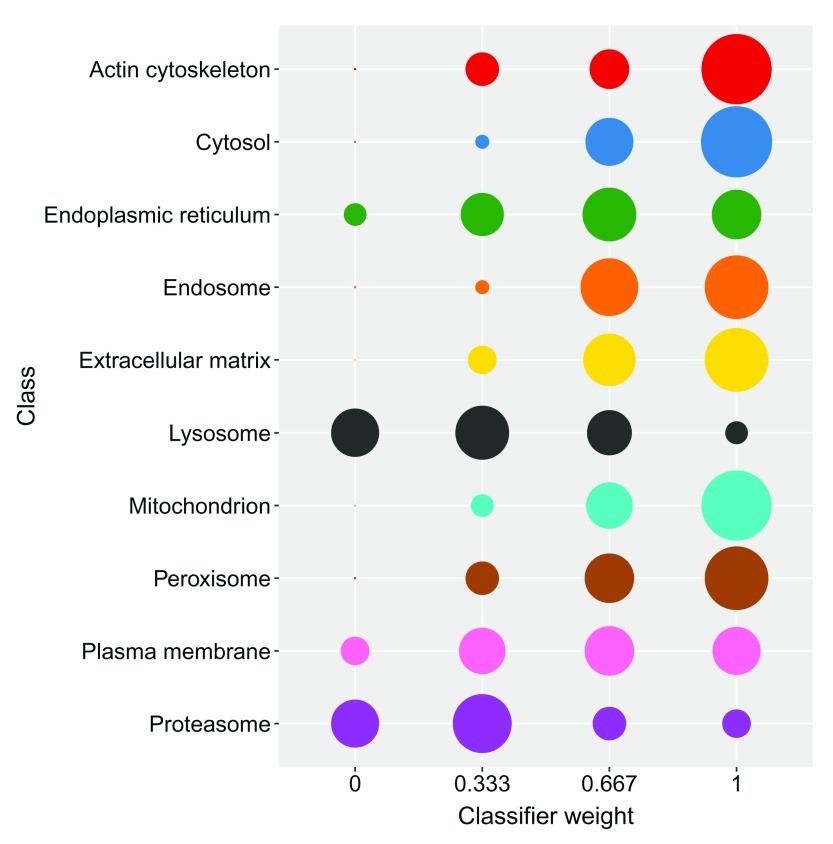
Visualisation of the transfer learning parameter optimisation procedure. Each row displays the frequency of observed weights (along the columns) for a specific sub-cellular class, with large dots representing higher observation frequencies.

The results of the optimisation can be visualised using the plot method for tlopt optimisation result (shown in Figure 20):



plot (tlopt)
                    


### TL classification

Looking at the bubble plot displaying the distribution of best weights over the 50 runs we find that for many of the subcellular niches a weight of 1 is most popular (i.e. use only primary hyperLOPIT data in classification), this is unsuprising as we already know the dataset is well resolved for these classes. We see that the most popular weights for the proteasome and lysosome tend to be towards 0, indicating that these niches are well-resolved in the Gene Ontology. This tells us that we would benefit from including auxiliary GO information in our classifier for these subcellular compartments. The plasma membrane weights are relatively equally spread between using hyperLOPIT and GO data. Using the getParams function we can return the best weights and then use this as input for the classification.

One of the benefits of the algorithm is the ability to manually select weights for each class. In the optimisation above, for time constraints, we removed the two ribosomal subunits and the two nuclear compartments, and therefore in the code chunk below when we extract the best parameters, these subcellular niches are not included. To include these 4 subcellular niches in the next classification step we must include them in the parameters. We define a weight of 1 for each of these niches, as we know they are well resolved in hyperLOPIT. We then re-order the weights according to getMarkerClasses and perform the classification using the function knntlClassification.



## best parameters for the 10 classes

(bestpar <- getParams (tlopt))


##    Actin cytoskeleton                Cytosol   Endoplasmic reticulum
##             1.0000000              1.0000000               0.6666667
##              Endosome   Extracellular matrix                Lysosome
##             1.0000000              1.0000000               0.3333333
##         Mitochondrion             Peroxisome         Plasma membrane
##             1.0000000              1.0000000               0.6666667
##            Proteasome
##             0.3333333


## add weights for classes not included in the optimisation

otherweights <- rep ( 1 , 4 )

names (otherweights) <- c ( "40S Ribosome" , "60S Ribosome" ,
                            
                        "Nucleus - Chromatin" ,
                            
                        "Nucleus - Non-chromatin" )

(bestpar <- c (bestpar, otherweights))


##      Actin cytoskeleton                 Cytosol   Endoplasmic reticulum
##               1.0000000               1.0000000               0.6666667
##                Endosome    Extracellular matrix                Lysosome
##               1.0000000               1.0000000               0.3333333
##           Mitochondrion              Peroxisome         Plasma membrane
##               1.0000000               1.0000000               0.6666667
##              Proteasome            40S Ribosome            60S Ribosome
##               0.3333333               1.0000000               1.0000000
##     Nucleus - Chromatin Nucleus - Non-chromatin
##               1.0000000               1.0000000


## re-order classes

bestpar <- bestpar[ getMarkerClasses (hl)]


## Do the classification

hl <- knntlClassification (hl, gocc, bestTheta = bestpar, k = c ( 3 , 3 ))
                    


The results from the classification results and associated scores are appended to the fData slot and named knntl and knntl.scores respectively. Results can be visualised using plot2D, scores assessed and cutoffs calculated using the classify app in pRolocVis, predictions obtained using getPredictions in the same way as demonstrated above for the SVM classifier.

In *pRoloc*’s transfer learning vignette, we demonstrate how to use imaging data from the Human Protein Atlas ^26^ via the *hpar* package ^27^ as an auxiliary data source.

## Unsupervised machine learning

In *pRoloc* there is functionality for unsupervsied machine learning methods. In unsupervised learning, the training data consists of a set of input vectors e.g. protein profiles, ignoring the information about the class label e.g. localisation, other than for annotation purposes. The main goal in unsupervised learning is to uncover groups of similar features within the data, termed clustering. Ordination methods such as principal components analysis (PCA) also fall into the category of unsupervised learning methods, where the data can be projected from a high-dimensional space down to two or three dimensions.

**Figure 21.**
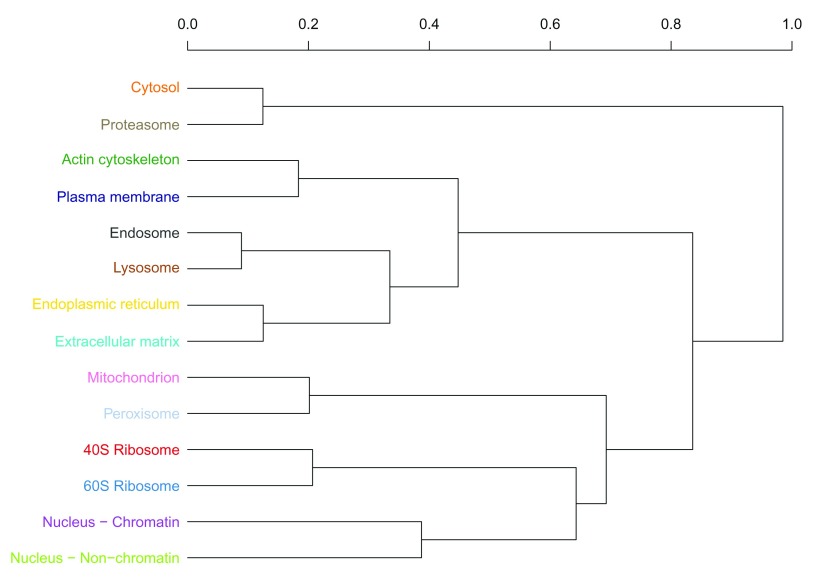
Hierarchical clustering of the average marker profiles summarising the relation between organelles profiles.

As described and demonstrated already above, PCA is a valuable and powerful method for data visualisation and quality control. Another application uses hierarchical clustering to summarise the relation between marker proteins using the mrkHClust function, where the euclidean distance between average class-specific profiles is used to produce a dendrogramme describing a simple relationship between the sub-cellular classes ( Figure 21). The mrkHClust uses the same defaults as all other function, using the markers feature variable to define marker proteins. In the code chunk, we adapt the figure margins to fully display the class names.



par ( mar = c ( 15 , 4 , 1 , 0 )) ## set figure margin

mrkHClust (hl)
                


We generally find supervised learning more suited to the task of protein localisation prediction in which we use high-quality curated marker proteins to build a classifier, instead of using an entirely unsupervised approach to look for clusters and then look for enrichment of organelles and complexes. In the latter we do not make good use of valuable prior knowledge, and in our experience unsupervised clustering can be extremely difficult due to (i) the loose definition of what constitutes a cluster (for example whether it is defined by the quantitative data or the localisation information), (ii) the influence of the algorithm assumption on the cluster identification (for example parametric or non-parametric) and (iii) poor estimates of the number of clusters that may appear in the data.

## Writing and exporting data

An MSnSet can be exported from R using the write.exprs function. This function writes the expression values to a text-based spreadsheet. The fcol argument can be used to specify which featureData columns (as column names, column number or logical) to append to the right of the expression matrix.

In the below code chunk we write the hl object to a csv file. The file argument is used to specify the file path, the sep argument specifies the field separator string, here we use a comma, finally as we want to write all the information in the featureData to the file, as well as the expression data, we specify fvarLabels(hl), that returns all feature variable names, and write the resulting data to the file "hl.csv".



write.exprs (hl, file = "hl.csv" , sep = "," , fcol = fvarLabels (hl))
                


Exporting to a spreadsheet however loses a lot of important information, such as the processing data, and the sample metadata in the *phenoData* slot. Other objects, such as parameters from the machine learning optimisation, cannot be represented as tabular data. To directly serialise R objects to disk, on can use the standard save function, and later reload the object using load. For example, to save and then re-load the parameters from the SVM optimisation,



## To save the parameters as an R object

save (params, file = "svmparams.rda" )


## To re-load after saving

load ( file = "svmparams.rda" )
                


## Session information and getting help

The function sessionInfo provides a summary of all packages and versions used to generate this document. This enables us to record the exact state of our session that lead to these results. Conversely, if the script stops working or if it returns different results, we are in a position to re-generate the original results using the adequate software versions and retrace changes in the software that lead to failure and/or different results.



sessionInfo ()
			

## R Under development (unstable) (2018-04-02 r74505)
## Platform: x86_64-pc-linux-gnu (64-bit)
## Running under: Ubuntu 14.04.5 LTS
##
## Matrix products: default
## BLAS: /usr/lib/atlas-base/atlas/libblas.so.3.0
## LAPACK: /usr/lib/lapack/liblapack.so.3.0
##
## locale:
##  [1] LC_CTYPE=en_GB.UTF-8       LC_NUMERIC=C
##  [3] LC_TIME=en_GB.UTF-8        LC_COLLATE=en_GB.UTF-8
##  [5] LC_MONETARY=en_GB.UTF-8    LC_MESSAGES=en_GB.UTF-8
##  [7] LC_PAPER=en_GB.UTF-8       LC_NAME=C
##  [9] LC_ADDRESS=C               LC_TELEPHONE=C
## [11] LC_MEASUREMENT=en_GB.UTF-8 LC_IDENTIFICATION=C
##
## attached base packages:
## [1] stats4    parallel  stats     graphics  grDevices utils     datasets
## [8] methods   base
##
## other attached packages:
##  [1] pRolocdata_1.19.0    pRoloc_1.21.0        MLInterfaces_1.61.1
##  [4] cluster_2.0.7-1      annotate_1.59.0      XML_3.98-1.11
##  [7] AnnotationDbi_1.43.1 IRanges_2.15.13      S4Vectors_0.19.5
## [10] MSnbase_2.7.1        ProtGenerics_1.13.0  BiocParallel_1.15.3
## [13] mzR_2.15.1           Rcpp_0.12.17         Biobase_2.41.0
## [16] BiocGenerics_0.27.0  xtable_1.8-2         BiocStyle_2.9.2
## [19] knitr_1.20
##
## loaded via a namespace (and not attached):
##   [1] backports_1.1.2       plyr_1.8.4            igraph_1.2.1
##   [4] lazyeval_0.2.1        splines_3.6.0         ggvis_0.4.3
##   [7] crosstalk_1.0.0       ggplot2_2.2.1         digest_0.6.15
##  [10] foreach_1.4.4         BiocInstaller_1.31.1  htmltools_0.3.6
##  [13] viridis_0.5.1         gdata_2.18.0          magrittr_1.5
##  [16] memoise_1.1.0         doParallel_1.0.11     sfsmisc_1.1-2
##  [19] limma_3.37.1          recipes_0.1.2         gower_0.1.2
##  [22] rda_1.0.2-2           dimRed_0.1.0          lpSolve_5.6.13
##  [25] prettyunits_1.0.2     colorspace_1.3-2      blob_1.1.1
##  [28] dplyr_0.7.5           RCurl_1.95-4.10       hexbin_1.27.2
##  [31] genefilter_1.63.0     bindr_0.1.1           impute_1.55.0
##  [34] DRR_0.0.3             survival_2.42-3.1     iterators_1.0.9
##  [37] glue_1.2.0            gtable_0.2.0          ipred_0.9-6
##  [40] zlibbioc_1.27.0       ddalpha_1.3.3         kernlab_0.9-26
##  [43] prabclus_2.2-6        DEoptimR_1.0-8        abind_1.4-5
##  [46] scales_0.5.0          vsn_3.49.0            mvtnorm_1.0-7
##  [49] DBI_1.0.0             viridisLite_0.3.0     progress_1.1.2
##  [52] magic_1.5-8           foreign_0.8-70        bit_1.1-13
##  [55] proxy_0.4-22          mclust_5.4            preprocessCore_1.43.0
##  [58] lava_1.6.1            prodlim_2018.04.18    sampling_2.8
##  [61] htmlwidgets_1.2       httr_1.3.1            threejs_0.3.1
##  [64] FNN_1.1               RColorBrewer_1.1-2    fpc_2.1-11
##  [67] modeltools_0.2-21     pkgconfig_2.0.1       flexmix_2.3-14
##  [70] nnet_7.3-12           caret_6.0-79          tidyselect_0.2.4
##  [73] rlang_0.2.0           reshape2_1.4.3        later_0.7.2
##  [76] munsell_0.4.3         mlbench_2.1-1         tools_3.6.0
##  [79] msdata_0.21.0         RSQLite_2.1.1         pls_2.6-0
##  [82] broom_0.4.4           geometry_0.3-6        evaluate_0.10.1
##  [85] stringr_1.3.1         mzID_1.19.0           yaml_2.1.19
##  [88] ModelMetrics_1.1.0    bit64_0.9-7           robustbase_0.93-0
##  [91] randomForest_4.6-14   purrr_0.2.4           dendextend_1.8.0
##  [94] bindrcpp_0.2.2        nlme_3.1-137          whisker_0.3-2
##  [97] mime_0.5              RcppRoll_0.2.2        biomaRt_2.37.1
## [100] compiler_3.6.0
##  [ reached getOption("max.print") -- omitted 38 entries ]
                


We also recommend that users regularly update the packages as well as the R itself. This can be done with the biocLite function.



library ( "BiocInstaller" )

biocLite ()
                


It is always important to include session information details along with a short reproducible example highlighting the problem or question at hand.

The source of this document, including the code necessary to reproduce the analyses and figures is available in a public manuscript repository on GitHub ^18^.

## Data availability

The data referenced by this article are under copyright with the following copyright statement: Copyright: © 2018 Breckels LM et al.

Data associated with the article are available under the terms of the Creative Commons Zero "No rights reserved" data waiver (CC0 1.0 Public domain dedication). 

The software and data presented in this workflow are part of the Bioconductor project. Version numbers for all packages used are shown in the Session information section.

The source of this document, including the code necessary to reproduce the analyses and figures is available at: https://github.com/lmsimp/bioc-pRoloc-hyperLOPIT-workflow/ ^18^. An archived version as at the time of publication is available at: DOI https://zenodo.org/record/1256018 ^28^.

## Appendix

### Novelty detection results

To load the pre-computed novelty detection results please execute the following lines of code

## Specifying the path to the PD results

f0 <- dir (extdatadir, full.names = TRUE,
            pattern = "bpw-pdres.rds" )

## Reading the PD results

pdres <- readRDS (f0)

## Adding the PD results to the hl MSnSet

hl <- addMarkers (hl, pdres, mcol = "pd", verbose = FALSE )

### SVM optimisation

To load the results of the SVM optimisation

## Specifying the path to the SVM optimisation results

svmf <- dir (extdatadir, full.names = TRUE ,
            pattern = "bpw-svmopt.rds" )

## Reading the results

params <- readRDS (svmf)

### Transfer learning results

To load the GO CC dataset

gofile <- dir (extdatadir, full.names = TRUE ,
            pattern = "bpw-gocc.rds" )
gocc <- readRDS (gofile)

To load the results of the transfer learning

tlfile <- dir (extdatadir, full.names = TRUE ,
            pattern = "bpw-tlopt.rds" )
tlopt <- readRDS (tlfile)
