# Supplementary material for: A Bioconductor workflow for processing and analysing spatial proteomics data
Source: F1000Res. 2018 Jul 3;5:2926. Originally published 2016 Dec 28. [Version 2] doi: 10.12688/f1000research.10411.2 (PMC6053703; doi:10.12688/f1000research.10411.2)
Supplement: Peer review history

# Peer review: Referee response for version 2

**DOI:** 10.5256/f1000research.16436.r35696

**Published:** 2018-07-16

**Authors:** Daniel J. Stekhoven

**Affiliations:** 1 NEXUS Personalized Health Technologies, ETH Zurich, Zürich, Switzerland

The revised manuscript of Breckels *et al. *addresses all my previously stated comments. The inclusion of code snippets in the appendix to fully reproduce the results from the authors adds to the overall very high credibility and quality of the article. It can be seen as a poster child article for reproducible research.

 For not finding the appropriate, computationally costly files in the first review online, I would like to excuse myself.

 Any other issues will be dealt with through the corresponding developer channels.

I have read this submission. I believe that I have an appropriate level of expertise to confirm that it is of an acceptable scientific standard.

# Peer review: Referee response for version 1

**DOI:** 10.5256/f1000research.11217.r18815

**Published:** 2017-01-18

**Authors:** Daniel J. Stekhoven

**Affiliations:** 1 NEXUS Personalized Health Technologies, ETH Zurich, Zürich, Switzerland

*Breckels et al. *have written a very nice piece on analysing appropriate proteomics data for subcellular localisation. I particularly like the " *workshop characteristics*" of the text. Which allows a novice, but interested reader to work through the analysis stepwise and reproduce the results described therein. The authors took great care in keeping this ideal up during their text and this is also where I have put my greatest reservation to the manuscript in its present form - since a reader cannot work through the code presented in the manuscript, since there at at least two situations where a readily available HPC and quite some time is required. This kind of leaves a dent in my impression - however, given this can be resolved as well as some typos - the workflow report is superb.

**Major comments:**

- Next to reducing the dimensions of data for visualisation, PCA also offers a way to understand how the variability is distributed across the multidimensional data by providing linear combinations of the variables which then constitute the actual PCs. On that note it would be nice to mention this in Visualising markers section on page 16, where PC7 explains not much variability but due to the correct weighing of the variables we do get a separation between mitochondrial and peroxisome. This then can be further motivated with Figure 9 - where we probably can see that the weights for the fractions where the two localisations differ are larger than otherwise.
- I was unable to reproduce Figure 13 comparing the two MSnSets. While I was able to look at each set separately using pRolocVis(hllst@x[[I]]), where i is 1 or 2, I only got an error using the code from the manuscript:
  > pRolocVis(hllst, app=“compare”)
  Subsetting MSnSetList to their common feature names
  5032 features in common
  Remapping data to the same PC space
  Error in (function (od, vd)  : 
    object and replacement value dimnames differ
  Error in pRolocVis_compare(object, ...) : object 'idDT' not found
  When using ‘remap=FALSE’ it actually works, but since this makes barely sense it is of no use - but just as a hint at debugging it.
- You really need to make the results from the phenoDisco classification available too. It is super disappointing that one cannot continue reproducing the code from page 23 on, because it takes 24 hours to compute it using 40 cores…
- The above comment is of course also true for the KNN TL Optimisation on page 33 - this needs to be downloadable, since not everyone has access to Cambridge’s HPC and probably even less have 76 hours to spare.
- Your comment on the increase suitability of classification instead of clustering (when additional information on classes is available) at the bottom of page 35 could be more pronounced - for educational reasons.

**Minor comments:**

- I was not able to naïvely reproduce the workflow from the R commands in the article due to an error installing *pRolocdata* on a Windows machine. On OS X it was smooth.
- On page 10 line 2 there is a ‘to’ missing.
- I never came across the verb *imputate* in the context of missing values, I guess the proper term is impute.
- On page 11 the *image2* function is called after the *filterNA* function a couple of lines above. This however would result in an only black heat map (since there are no more missing). The *image2* function should be called before the *filterNA* function. Since the reader does not see the chunk options, it could be puzzling.
- For completeness sake there should also be an *install.packages(c(“hexbin”, “rgl”))* somewhere to generate the second PCA-plot and the 3D plot. Moreover, Mac users will need to install *xquartz* to use *rgl* properly.
- On page 14 the plotting code chunk is off track - in the middle of the marker sets output.
- On page 18: …wanted to highlight a proteins with the… -> lose the *a* and later in the sentence there is a ‘ *create a*’ too many.
- Direct comparisons of individual channels in replicated experiments *do* not provide…
- You may want to consider adding a *layout(1)* or similar, after changing the *mfrow* argument of the parameters to accommodate 2 panels, such that the uncanny reader does not get confused.
- I would prefer links to referred sections of the text, but that may be personal taste…
- Page 23: One should note that the decreasing the GS, and increasing the … at least one the too many, probably two.
- On page 25: We find the general tendancy to be that it is not the choice … tendency?
- On page 28 you refer to ‘…the code chunk below…’ for Figure 17, however, the following code chunk is generating Figure 16 (which is above and btw not referenced in the text). Maybe force your figures a little to float where you want them/refer to them.
- On page 28: …by extracting the median or 3rd quantile score per organelle… do you mean quartile? Otherwise I do not follow.
- On page 32: …package to query the relevent database … relevant?
- On page 32 - there is something wrong with this sentence: *To remove the 4 classes and create a new column of markers in the feature data called tlmarkers to use for the analysis*:
- On page 34: From examining the parameter seach plots as described in section Optimisation… search!
- On page 36: …and later reload the object using save. -> that would be ‘load’ then!
- On page 38 -  I fully agree with the following sentence, but right after the updating comment it kind of seems ‘ *misplaced*’? Maybe add a title like ‘ *Getting help*’?
*It is always important to include session information details along with a short reproducible example highlighting the problem or question at hand.*

I have read this submission. I believe that I have an appropriate level of expertise to confirm that it is of an acceptable scientific standard, however I have significant reservations, as outlined above.

# Response

**Published:** 2018-05-23

**Authors:** Laurent Gatto

**Affiliations:** University of Cambridge, UK

Thank you for your comments. Please find our responses to these inset below.

*Next to reducing the dimensions of data for visualisation, PCA also offers a way to understand how the variability is distributed across the multidimensional data by providing linear combinations of the variables which then constitute the actual PCs. On that note it would be nice to mention this in Visualising markers section on page 16, where PC7 explains not much variability but due to the correct weighing of the variables we do get a separation between mitochondrial and peroxisome. This then can be further motivated with Figure 9 - where we probably can see that the weights for the fractions where the two localisations differ are larger than otherwise.*

 We have added a paragraph to the 'Visualising markers' section of the manuscript reiterating the purpose of PCA and motivating the choice of looking at PC's 1 and 7. Figure 9 now follows on from this (now Figure 8), along with the corresponding code and an explanation of the `plotDist` function.

*I was unable to reproduce Figure 13 comparing the two MSnSets. While I was able to look at each set separately using `pRolocVis(hllst@x[[I]])`, where i is 1 or 2, I only got an error using the code from the manuscript. When using `remap=FALSE` it actually works, but since this makes barely sense it is of no use - but just as a hint at debugging it.*

 We can not reproduce this error. Have you updated to the latest version of R and the latest version of `pRolocGUI`? If you still get this error message could you please post this as an issue (https://github.com/ComputationalProteomicsUnit/pRolocGUI/issues) on the `pRolocGUI` Github page along with your `sessionInfo()` and we will certainly attempt to solve this.

*You really need to make the results from the phenoDisco classification available too. It is super disappointing that one cannot continue reproducing the code from page 23 on, because it takes 24 hours to compute it using 40 cores?*

 The results are already available as a `RDS` file and stored in `pRolocdata` for users. This is what is called in the manuscript under the hood:

 ```

 f0 <- dir(extdatadir, full.names = TRUE, pattern = "bpw-pdres.rds")

 pdres <- readRDS(f0)

 hl <- addMarkers(hl, pdres, mcol = "pd", verbose = FALSE)

 ```

 We have made this code available in the manuscript in an appendix so users can continue to produce the exact plots as they see in this workflow.

*The above comment is of course also true for the KNN TL Optimisation on page 33 - this needs to be downloadable, since not everyone has access to Cambridge's HPC and probably even less have 76 hours to spare.*

 The same as for the `phenoDisco` analysis and `svm`, the TL results are stored as a `RDS` in `pRolocdata` and are loaded in the background. We have added the code required to the appendix so that users can load the results directly.

*Your comment on the increase suitability of classification instead of clustering (when additional information on classes is available) at the bottom of page 35 could be more pronounced - for educational reasons.*

 To address the above comment on suitability we have added a few additional points on the challenges of using clustering for this type of data.

 We generally find supervised learning more suited to the task of protein localisation prediction in which we use high-quality curated marker proteins to build a classifier, instead of using an entirely unsupervised approach to look for clusters and then look for enrichment of organelles and complexes. In the latter we do not make good use of valuable prior knowledge, and in our experience unsupervised clustering can be extremely difficult due to (i) the loose definition of what constitutes a cluster (for example whether it is defined by the quantitative data or the localisation information), (ii) the influence of the algorithm assumption on the cluster identification (for example parametric or non-parametric) and (iii) poor estimates of the number of clusters that may appear in the data.

*I was not able to naively reproduce the workflow from the R commands in the article due to an error installing pRolocdata on a Windows machine. On OS X it was smooth.*

 We didn't experience any Windows-specific problems. If you re-try the installation and please let us know if you still have any issues by opening an issue (https://github.com/lgatto/pRolocdata/issues) or by posting this issue on the Bioconductor Support site (https://support.bioconductor.org).

On page 10 line 2 there is a *to* missing.

 In the version have, we currently can't find the missing 'to'.

*I never came across the verb imputate in the context of missing values, I guess the proper term is impute.*

 This has been changed to read "We can impute missing data..."

*On page 11 the image2 function is called after the filterNA function a couple of lines above. This however would result in an only black heat map (since there are no more missing). The image2 function should be called before the filterNA function. Since the reader does not see the chunk options, it could be puzzling.*

 This was an editing mistake and has now been rectified.

*For completeness sake there should also be an install.packages(c("hexbin", "rgl")) somewhere to generate the second PCA-plot and the 3D plot. Moreover, Mac users will need to install xquartz to use rgl properly.*

 A footnote has been added here to tell users that the package `rgl` may need to be installed with `install.packages("rgl")` and mac users may need to install xquartz if it's not already installed. LG: this one is still missing.

*On page 14 the plotting code chunk is off track - in the middle of the marker sets output.*

 This has now been rectified.

*On page 18: ...wanted to highlight a proteins with the ... -> lose the a and later in the sentence there is a 'create a' too many.*

 These typos have been rectified.

*Direct comparisons of individual channels in replicated experiments do not provide?*

*You may want to consider adding a layout(1) or similar, after changing the mfrow argument of the parameters to accommodate 2 panels, such that the uncanny reader does not get confused.*

 We would prefer to keep the code as it is and not introduce more noise with calls to other functions such as `layout`. The workflow is not aimed at teaching R. Users should have some basic knowledge of R before tackling this tutorial.

*I would prefer links to referred sections of the text, but that may be personal taste?*

 This is a comment for F1000. We cannot control the linking of sections in the final version.

*Page 23: One should note that the decreasing the GS, and increasing the ...at least one the too many, probably two.*

 We have reworded this sentence as requested.

*On page 25: We find the general tendancy to be that it is not the choice ...tendency?*

 This typo has been rectified.

*On page 28 you refer to '...the code chunk below...' for Figure 17, however, the following code chunk is generating Figure 16 (which is above and btw not referenced in the text). Maybe force your figures a little to float where you want them/refer to them.*

 We have now referenced Figure 16 in the text and made sure that the code chunks and figures follow inline where they are referenced in the text.

*On page 28: ...by extracting the median or 3rd quantile score per organelle? do you mean quartile? Otherwise I do not follow.*

 Thank you, yes this is a typo and has now been changed to 'quartile'.

On page 32: package to query the relevent database *relevant*

 This typo has been rectified.

*On page 32 - there is something wrong with this sentence: To remove the 4 classes and create a new column of markers in the feature data called tlmarkers to use for the analysis:*

 This sentence is not needed here and so it has now been removed as it essentially reiterates what is said in the above paragraph.

*On page 34: From examining the parameter seach plots as described in section Optimisation... search!*

 This typo has been rectified.

*On page 36: and later reload the object using save. -> that would be `load` then!*

 This typo has been rectified.

*On page 38 - I fully agree with the following sentence, but right after the updating comment it kind of seems misplaced? Maybe add a title like Getting help?*

 We have changed the title of this section to 'Session information and getting help' to clarify this section of the tutorial.

 A pdf version of our replies is also available here.

# Peer review: Referee response for version 1

**DOI:** 10.5256/f1000research.11217.r18813

**Published:** 2017-01-11

**Authors:** Leonard J. Foster

**Affiliations:** 1 Michael Smith Laboratories -Centre for High-Throughput Biology (CHiBi), Department of Biochemistry and Molecular Biology, University of British Columbia (UBC), Vancouver, BC, Canada

This manuscript describes a Bioconductor workflow for analyzing subcellular proteomics data. It is very detailed and comprehensive and will be useful for others in the field. A few comments:

- Some clearer statement early on would help to clarify for readers what types of data this works with. I know that the authors indicate that the example they use is 10-plex TMT and that it can be used with label-free or other labels, but that is not what I am referring to. Rather, structure of the experiment. That is, that one needs systematic quantitative data on all the different relevant fractions from a cell, as opposed to someone who perhaps did a differential centrifugation experiment to isolate a couple fractions and then wants to apply this (my understanding is that this latter example would not be usable).
- How do the authors recommend collapsing replicates? This could be covered in the section dedicated to the Compare function. Two replicates will (almost) never agree 100% so how are discrepancies handled?

I have read this submission. I believe that I have an appropriate level of expertise to confirm that it is of an acceptable scientific standard.

# Response

**Published:** 2018-05-23

**Authors:** Laurent Gatto

**Affiliations:** University of Cambridge, UK

*This manuscript describes a Bioconductor workflow for analyzing subcellular proteomics data. It is very detailed and comprehensive and will be useful for others in the field.*

 Many thanks for your comments, we have responded to them inset below.

*Some clearer statement early on would help to clarify for readers what types of data this works with. I know that the authors indicate that the example they use is 10-plex TMT and that it can be used with label-free or other labels, but that is not what I am referring to. Rather, structure of the experiment. That is, that one needs systematic quantitative data on all the different relevant fractions from a cell, as opposed to someone who perhaps did a differential centrifugation experiment to isolate a couple fractions and then wants to apply this (my understanding is that this latter example would not be usable).*

 From a purely technical point of view, we need a data matrix with features (typically proteins or protein groups) along the rows and sub-cellular fractions along the columns. Given the requirement for complete (or near-complete) quantitative vectors along all fractions to assure best results, a data set that would only contain one quantitation value per fraction would not work. However, separation using differential centrifugation, or any separation that generates organelle-specific separation profiles is a good fit for pRoloc. For instance, the type of data generated by methods such as described in Itzhak et al. (2016) are well fitted for our software:

 > library(pRoloc)

 > library(pRolocdata)

 > data(itzhak2016stcSILAC)

 > ## 6 combined replicates of 5 fractions each

 > dim(itzhak2016stcSILAC)

 [1] 5265   30

 > plot2D(itzhak2016stcSILAC)

 (see outout here)

*How do the authors recommend collapsing replicates? This could be covered in the section dedicated to the Compare function. Two replicates will (almost) never agree 100% so how are discrepancies handled?*

 Currently, we recommend to visualise different replicates on their own, to confirm that they are of sufficient quality, and then combine them, retaining the proteins that have been quantified over all replicatedd experiments. This allows to obtain localisation information over all replicated data. We however do not explicitly assess the variability using this approach. This could be done by analysing replicated independently and then compare the coherence of the classification results. Proteins that are only observed in some replicates could be rescued by repeating the analysis using only the relevant (possibly unique) replicate(s).
